# Supplementary material for: Structural basis for antiepileptic drugs and botulinum neurotoxin recognition of SV2A
Source: Nat Commun. 2024 Apr 18;15:3027. doi: 10.1038/s41467-024-47322-4 (PMC11026379; doi:10.1038/s41467-024-47322-4)
Supplement: Supplementary file 1 — Supplementary Information [file 41467_2024_47322_MOESM1_ESM.pdf]

Supplementary Information

Structural basis for antiepileptic drugs and botulinum neurotoxin recognition of SV2A

Atsushi Yamagata, Kaori Ito, Takehiro Suzuki, Naoshi Dohmae, Tohru Terada, Mikako Shirouzu

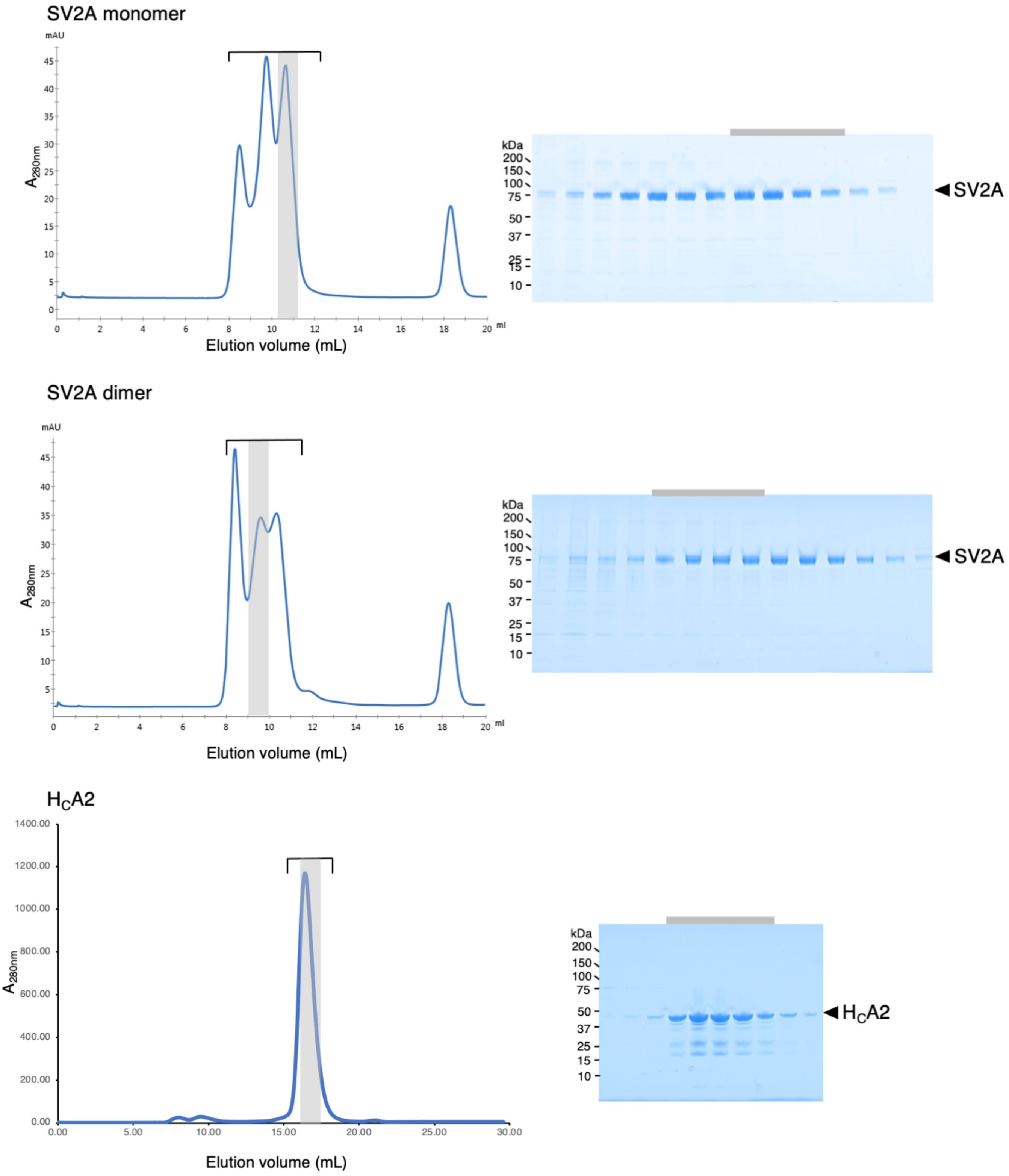

Supplementary Figure 1. Purification of SV2A and H$_C$A2

Size exclusion chromatography (SEC) profiles of SV2A in LMNG, that of SV2A in GDN, and that of H$_C$A2. The corresponding SDS-PAGE gels are shown right and the fractions in the shadowed area were pooled for futher study. Experiments were repeated more than twice independently with similar results

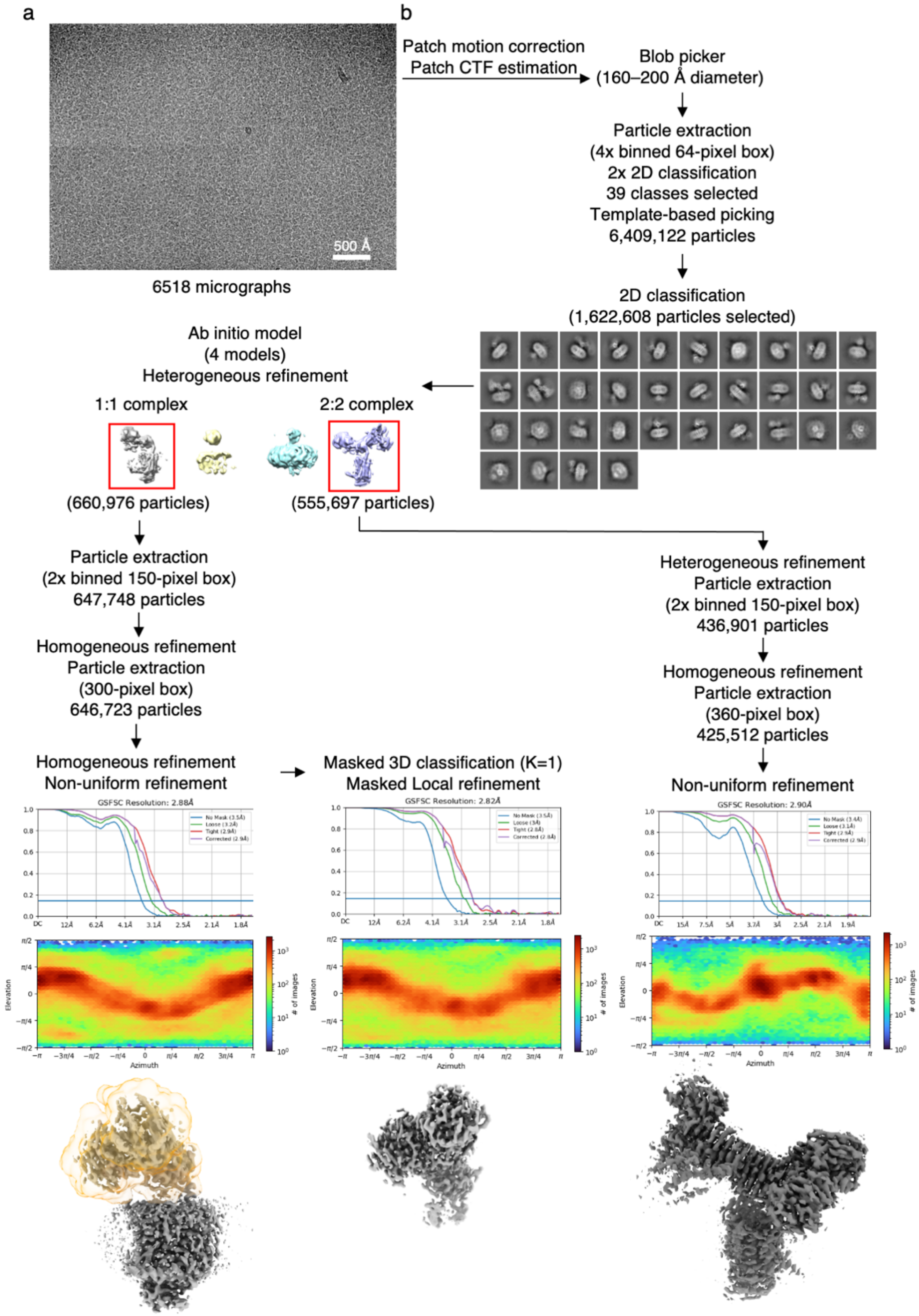

Supplementary Figure 2. Cryo-EM data and processing for the SV2A–H$_C$A2–LEV complex

a. A representative micrograph of the SV2A–H$_C$A2–LEV complex. We made at least two vitrified grids with similar particle images, and the micrographs were collected from a single grid. **b.** A flow chart for data processing and the final maps with Euler angle distribution plots.

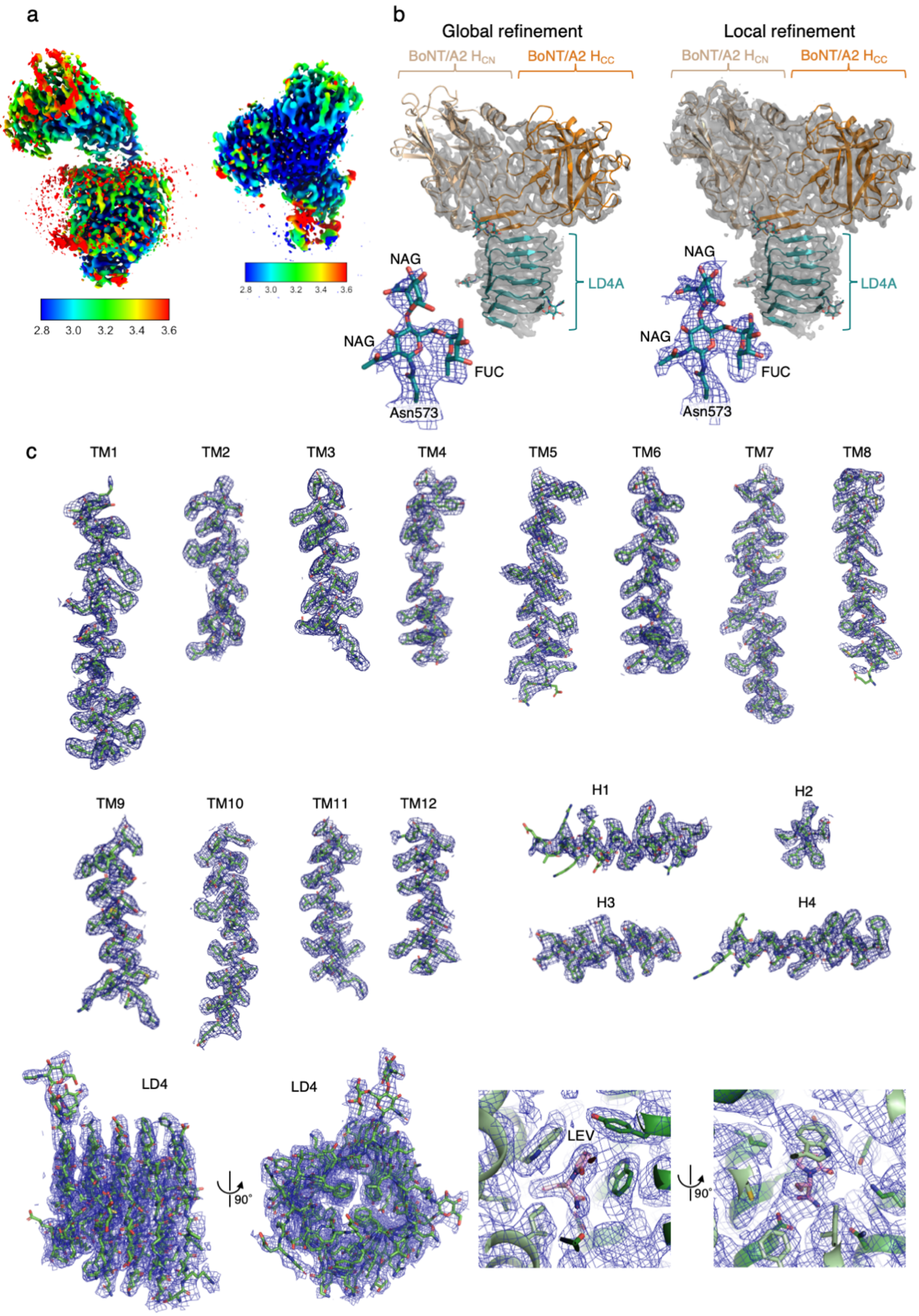

Supplementary Figure 3. Density maps for the SV2A–H$_C$A2–LEV complex

a. Local resolution maps of the global refinement map (SV2A–H$_C$A2–LEV) and the local refinement map (LD4A–H$_C$A2). **b.** The cryo-EM density of the global refinement map covering the LD4A–H$_C$A2 region, and that of the local refinement map. **c.** Cryo-EM densities for each secondary structure segments of SV2A TMD and those for LD4A. Cryo-EM densities for LEV with the surrounding residues, viewed from two different angles, are also shown.

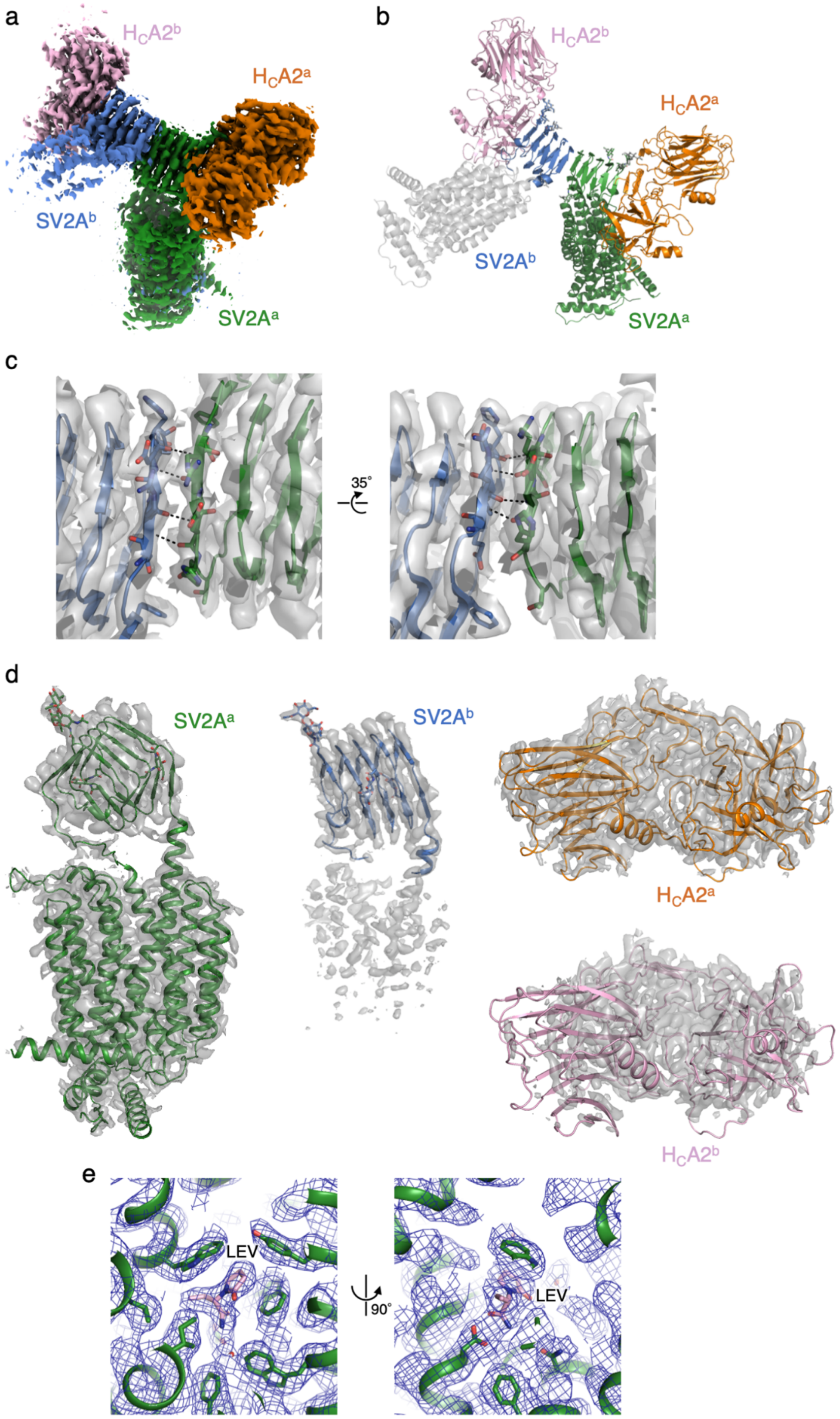

Supplementary Figure 4. Cryo-EM structure of the 2:2 assembly of the SV2A–H$_C$A2–LEV complex.

a. Cryo-EM map of the 2:2 assembly of the SV2A–H$_C$A2–LEV complex. One complex (SV2A^a–H$_C$A2^a) is colored in green and orange, respectively. The other complex (SV2A^b–H$_C$A2^b) is colored in blue and pink, respectively. Note that the TMD of the SV2A^b (blue) showed poor density. **b.** Ribbon representation of the 2:2 assembly of the SV2A–H$_C$A2–LEV complex. Coloring scheme is the same as that in **(a)**. The TMD of the SV2A^b (blue) is shown in gray, which was not involved in the final model. **c.** The interface of the LD4–LD4 interaction. **d.** Cryo-EM densities of two SV2A protomers and two H$_C$A2 protomers. **e.** Cryo-EM densities for LEV, viewed from two different angles.

```
hSV2A    MEEGFRDRAAFIRGAKDIAKEVKKHAAKKVVKGLDRVQDEYSRRSYSRFEEEDDDDDFPA 60
rSV2A    MEEGFRDRAAFIRGAKDIAKEVKKHAAKKVVKGLDRVQDEYSRRSYSRFEEEEDDDDFPA 60
mSV2A    MEEGFRDRAAFIRGAKDIAKEVKKHAAKKVVKGLDRVQDEYSRRSYSRFEEEDDDDDFPA 60
hSV2B    ---------------------------------------------MDDYKY-QDNYGGYA 14
rSV2B    ---------------------------------------------MDDYRY-RDNYEGYA 14
mSV2B    ---------------------------------------------MDDYRY-RDNYEGYA 14
hSV2C    MEDSYKDRTSLMKGAKDIAREVKKQTVKKVNQAVDRAQDEYTQRSYSRFQDEEDDDDYYP 60
rSV2C    MEDSYKDRTSLMKGAKDIAKEVKKQTVKKVNQAVDRAQDEYTQRSYSRFQDEDDDDDYYP 60
mSV2C    MEDSYKDRTSLMKGAKDIAKEVKKQTVKKVNQAVDRAQDEYTQRSYSRFQDEEDDDDYYP 60

hSV2A    PSDGYYRGEGTQDEEEGGASSDATEGHDEDDEIYEGEYQGIPRAESGGKGERMADGAPLA 120
rSV2A    PADGYYRGEGAQDEEEGGASSDATEGHDEDDEIYEGEYQGIPRAESGGKGERMADGAPLA 120
mSV2A    PADGYYRGEGAQDEEEGGASSDATEGHDEDDEIYEGEYQGIPRAESGGKGERMADGAPLA 120
hSV2B    PSDGYYRGNESNPEE--DAQSDVTEGHDEEDEIYEGEYQGIPHPDDVKAKQAKMAPSRMD 72
rSV2B    PNDGYYRGNEQNPEE--DAQSDVTEGHDEEDEIYEGEYQGIPHPDDVKSKQTKMAPSRAD 72
mSV2B    PSDGYYRSNEQNQEE--DAQSDVTEGHDEEDEIYEGEYQGIPHPDDVKSKQTKMAPSRAD 72
hSV2C    AGE-TYNGEANDD----EGSSEATEGHDEDDEIYEGEYQGIPSMNQAKDSIVSV------ 109
rSV2C    PGE-TYSGEANDD----EGSSEATEGHDEEDEIYEGEYQGIPSTNQGKDSIVSV------ 109
mSV2C    PGE-TYSGEVNDD----EGSSEATEGHDEEDEIYEGEYQGIPSTNQGKDSIVSV------ 109

                                H1                        TM1
hSV2A    GVRGGLSDGEGPPGGRGEAQRRKEREELAQQYEAILRECGHGRFQWTLYFVLGLALMADG 180
rSV2A    GVRGGLSDGEGPPGGRGEAQRRKDREELAQQYETILRECGHGRFQWTLYFVLGLALMADG 180
mSV2A    GVRGGLSDGEGPPGGRGEAQRRKDREELAQQYETILRECGHGRFQWTLYFVLGLALMADG 180
hSV2B    SLRGQ---------TDLMAERLEDEEQLAHQYETIMDECGHGRFQWILFFVLGLALMADG 123
rSV2B    GLRGQ---------ADLMAERMEDEEQLAHQYETIIDECGHGRFQWTLFFVLVLALMADG 123
mSV2B    GLGGQ---------ADLMAERMEDEEELAHQYETIIDECGHGRFQWTLFFVLGLALMADG 123
hSV2C    ---GQPKGDEYKDRRELESERRADEEELAQQYELIIQECGHGRFQWALFFVLGMALMADG 166
rSV2C    ---GQPKGDEYKDRRELESERRADEEELAQQYELIIQECGHGRFQWALFFVLGMALMADG 166
mSV2C    ---GQPKGDEYKDRRELESERRADEEELAQQYELIIQECGHGRFQWALFFVLGMALMADG 166

                                        TM2
hSV2A    VEVFVVGFVLPSAEKDMCLSDSNKGMLGLIVYLGMMVGAFLWGGLADRLGRRQCLLISLS 240
rSV2A    VEVFVVGFVLPSAEKDMCLSDSNKGMLGLIVYLGMMVGAFLWGGLADRLGRRQCLLISLS 240
mSV2A    VEVFVVGFVLPSAEKDMCLSDSNKGMLGLIVYLGMMVGAFLWGGLADRLGRRQCLLISLS 240
hSV2B    VEVFVVSFALPSAEKDMCLSSSKKGMLGMIVYLGMMAGAFILGGLADKLGRKRVLSMSLA 183
rSV2B    VEVFVVSFALPSAEKDMCLSSSKKGMLGLIVYLGMMAGAFILGGLADKLGRKKVLSMSLA 183
mSV2B    VEIFVVSFALPSAEKDMCLSSSKKGMLGLIVYLGMMAGAFILGGLADKLGRKKVLSMSLA 183
hSV2C    VEVFVVGFVLPSAETDLCIPNSGSGWLGSIVYLGMMVGAFFWGGLADKVGRKQSLLICMS 226
rSV2C    VEVFVVGFVLPSAETDLCIPNSGSGWLGSIVYLGMMVGAFFWGGLADKVGRKQSLLICMS 226
mSV2C    VEVFVVGFVLPSAETDLCIPNSGSGWLGSIVYLGMMVGAFFWGGLADKVGRKQSLLICMS 226

         TM3                   TM4
hSV2A    VNSVFAFFSSFVQGYGTFLFCRLLSGVGIGGSIPIVFSYFSEFLAQEKRGEHLSWLCMFW 300
rSV2A    VNSVFAFFSSFVQGYGTFLFCRLLSGVGIGGSIPIVFSYFSEFLAQEKRGEHLSWLCMFW 300
mSV2A    VNSVFAFFSSFVQGYGTFLFCRLLSGVGIGGSIPIVFSYFSEFLAQEKRGEHLSWLCMFW 300
hSV2B    VNASFASLSSFVQGYGAFLFCRLISGIGIGGALPIVFAYFSEFLSREKRGEHLSWLGIFW 243
rSV2B    INASFASLSSFVQGYGAFLFCRLISGIGIGGSLPIVFAYFSEFLSREKRGEHLSWLGIFW 243
mSV2B    INASFASLSSFVQGYGAFLFCRLISGIGIGGSLPIVFAYFSEFLSREKRGEHLSWLGIFW 243
hSV2C    VNGFFAFLSSFVQGYGFFLFCRLLSGFGIGGAIPTVFSYFAEVLAREKRGEHLSWLCMFW 286
rSV2C    VNGFFAFLSSFVQGYGFFLLCRLLSGFGIGGAIPTVFSYFAEVLAREKRGEHLSWLCMFW 286
mSV2C    VNGFFAFLSSFVQGYGFFLVCRLLSGFGIGGAIPTVFSYFAEVLAREKRGEHLSWLCMFW 286

         TM5                                  TM6
hSV2A    MIGGVYAAAMAWAIIPHYGWSFQMGSAYQFHSWRVFVLVCAFPSVFAIGALTTQPESPRF 360
rSV2A    MIGGVYAAAMAWAIIPHYGWSFQMGSAYQFHSWRVFVLVCAFPSVFAIGALTTQPESPRF 360
mSV2A    MIGGVYAAAMAWAIIPHYGWSFQMGSAYQFHSWRVFVLVCAFPSVFAIGALTTQPESPRF 360
hSV2B    MTGGLYASAMAWSIIPHYGWGFSMGTNYHFHSWRVFVIVCALPCTVSMVALKFMPESPRF 303
rSV2B    MTGGIYASAMAWSIIPHYGWGFSMGTNYHFHSWRVFVIVCALPATVSMVALKFMPESPRF 303
mSV2B    MTGGIYASAMAWSIIPHYGWGFSMGTNYHFHSWRVFVIVCALPATVSMVALKFMPESPRF 303
hSV2C    MIGGIYASAMAWAIIPHYGWSFSMGSAYQFHSWRVFVIVCALPCVSSVVALTFMPESPRF 346
rSV2C    MIGGIYASAMAWAIIPHYGWSFSMGSAYQFHSWRVFVIVCALPCVSSVVALTFMPESPRF 346
mSV2C    MIGGIYASAMAWAIIPHYGWSFSMGSAYQFHSWRVFVIVCALPCVSSVVALTFMPESPRF 346

         H2        H3
hSV2A    FLENGKHDEAWMVLKQVHDTNMRAKGHPERVFSVTHIKTIHQEDELIEIQSDTGTWYQRW 420
rSV2A    FLENGKHDEAWMVLKQVHDTNMRAKGHPERVFSVTHIKTIHQEDELIEIQSDTGTWYQRW 420
mSV2A    FLENGKHDEAWMVLKQVHDTNMRAKGHPERVFSVTHIKTIHQEDELIEIQSDTGTWYQRW 420
hSV2B    LLEMGKHDEAWMILKQVHDTNMRAKGTPEKVFTVSNIKTPKQMDEFIEIQSSTGTWYQRW 363
rSV2B    LLEMGKHDEAWMILKQVHDTNMRAKGTPEKVFTVSHIKTPKQMDEFIEIQSSTGTWYQRW 363
mSV2B    LLEMGKHDEAWMILKQVHDTNMRAKGTPEKVFTVSHIKTPKQMDEFIEIQSSTGTWYQRW 363
hSV2C    LLEVGKHDEAWMILKLIHDTNMRARGQPEKVFTVNKIKTPKQIDELIEIESDTGTWYRRC 406
rSV2C    LLEVGKHDEAWMILKLIHDTNMRARGQPEKVFTVNKIKTPKQIDELIEIESDTGTWYRRC 406
mSV2C    LLEVGKHDEAWMILKLIHDTNMRARGQPEKVFTVNKIKTPKQIDELIEIESDTGTWYRRC 406
```

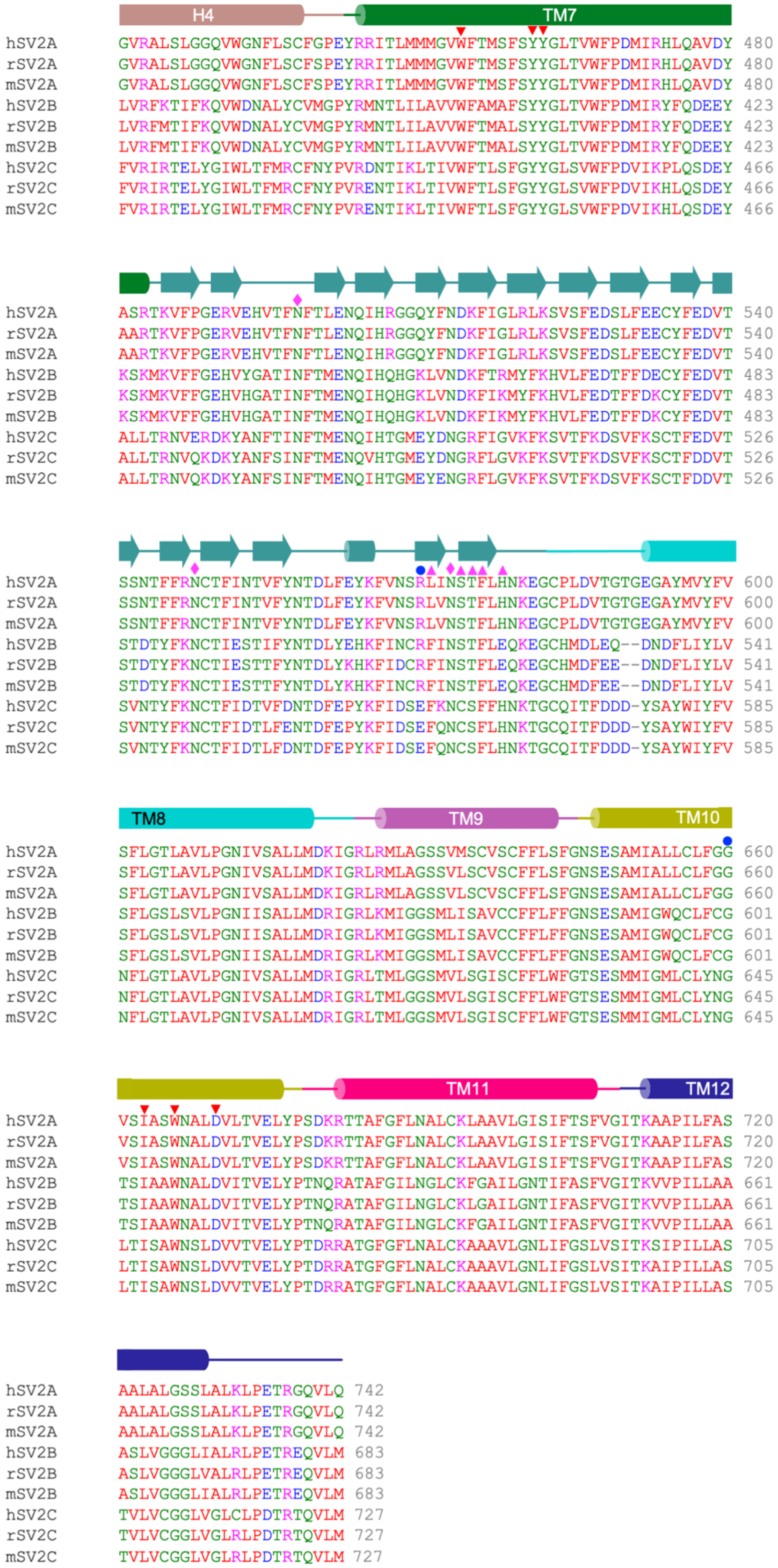

Supplementary Figure 5. Multiple sequence alignment of SV2 family.

The secondary structural elements are shown above the multiple sequence alignment. The residues involved in LEV/BRV-binding are shown in the red reverse triangle. The residues involved in H_CA2-binding and the glycan-attached Asn residues are shown in the magenta triangle and diamond, respectively. The human pathogenic mutation sites are shown in the blue circle. The residues involved in our hypothesized transport model are shown in cyan square, and the putative intrinsic flexible region in TM4 is highlighted in yellow.

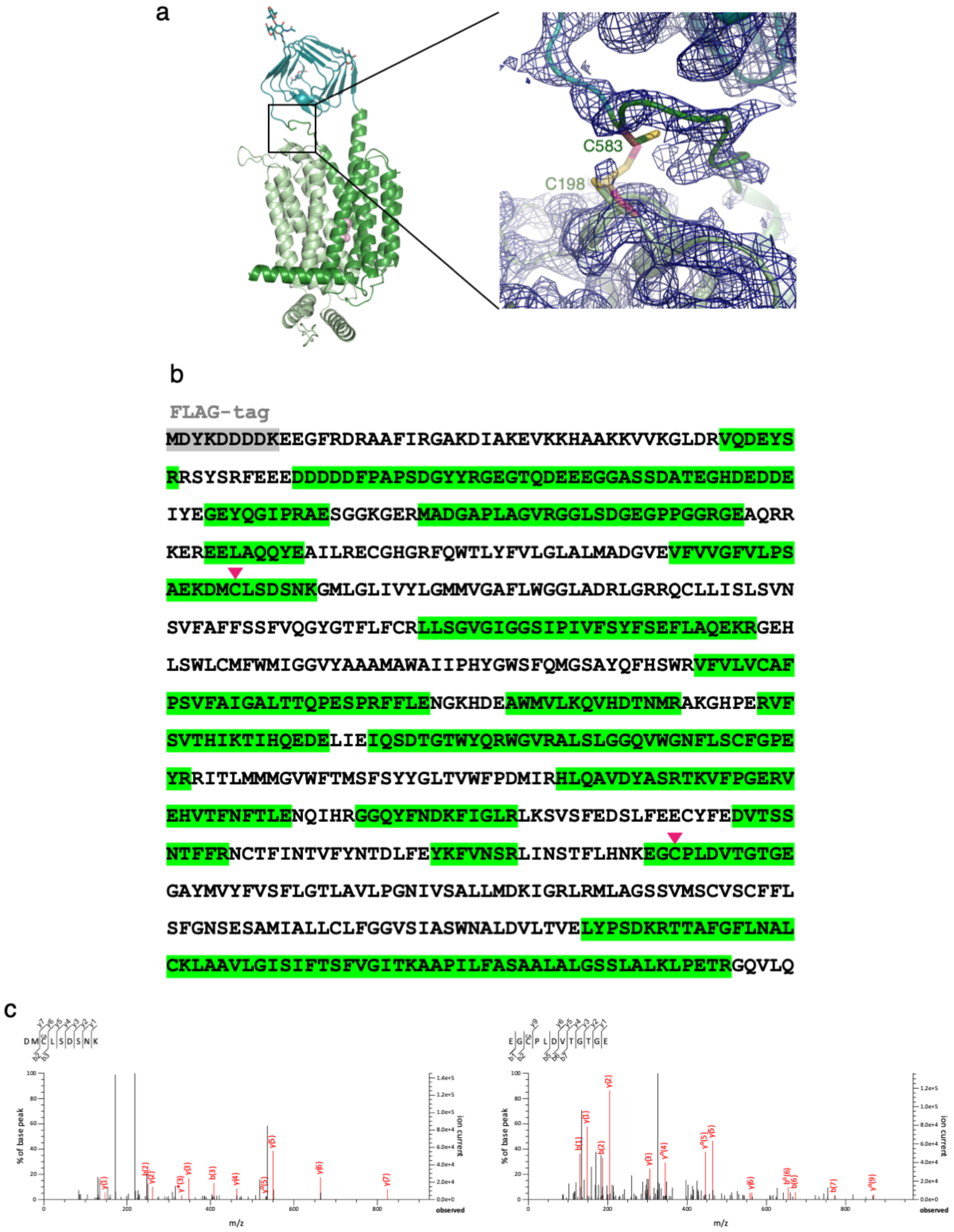

Supplementary Figure 6. Mass spectrometry analysis of SV2A.

a. The cryo-EM densities of the LD4–TM8 loop. Cys198 and Cys583 are close enough to form the disulfide bond. The tentative model of the disulfide bond is shown in hot pink. **b.** Coverage of the peptides identified by mass spectrometry analysis. Cys198 and Cys583 are highlighted in reverse triangles (hot pink). **d.** MSMS spectra of peptides containing Cys198 and Cys583. The peptides 'DMCLSDSNK' and 'EGCPLDVTGTGE' were identified with Mascot ion scores of 26 and 28, respectively.

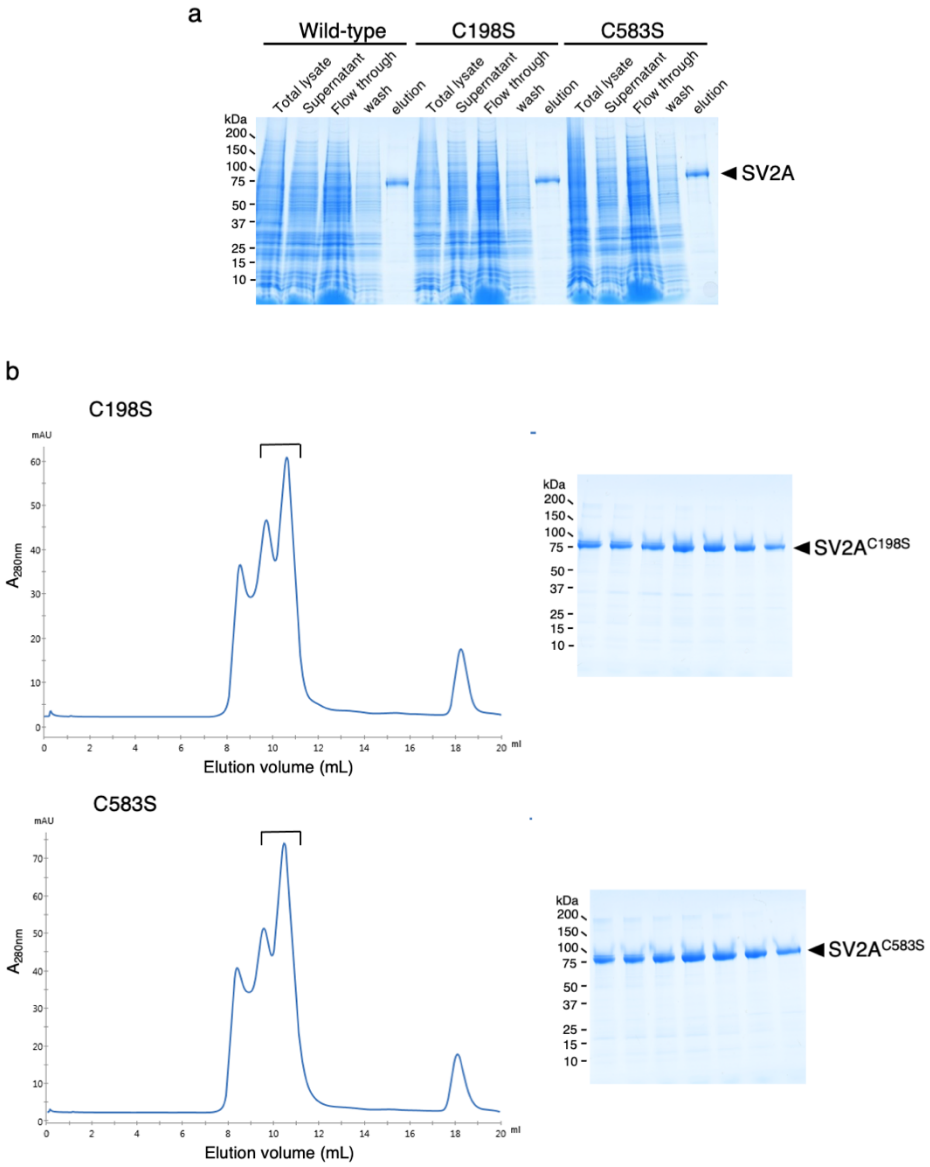

Supplementary Figure 7. Expression and SEC profiles of SV2A^{C198S} and SV2A^{C583S}

a. The expression and immunoaffinity purification of the wild-type, the C198S mutant, and the C583S mutant SV2As. The total lysate is the lysate after homogenization with LMNG. The supernatant is the cleared lysate after the ultracentrifugation of the total lysate. The flowthrough is that of the immunoaffinity purification using anti-FLAG M2 resin, and the elution is the final eluted sample after the wash. The mutant SV2As were expressed as well as the wild-type protein. **b.** SEC profiles of the C198S mutant and C583S mutant SV2A. The corresponding SDS-PAGE gels are shown right. Experiments were repeated twice independently with similar results.

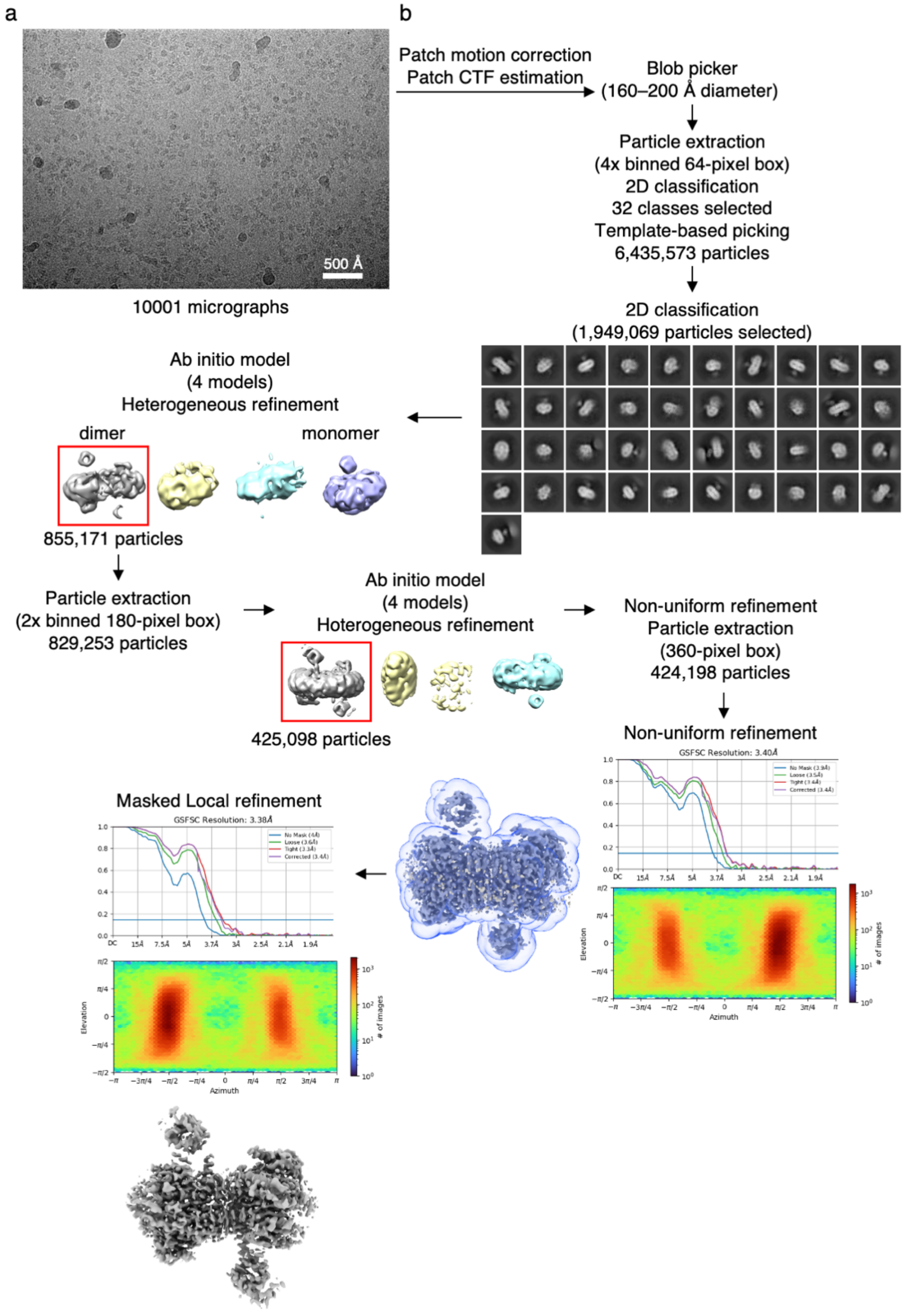

Supplementary Figure 8. Cryo-EM data and processing for the SV2A–LEV dimer

a. A representative micrograph of the SV2A–LEV dimer purified in the detergent GDN. We made at least two vitrified grids with similar particle images, and the micrographs were collected from a single grid. **b.** A flow chart for data processing and the final maps with Euler angle distribution plots.

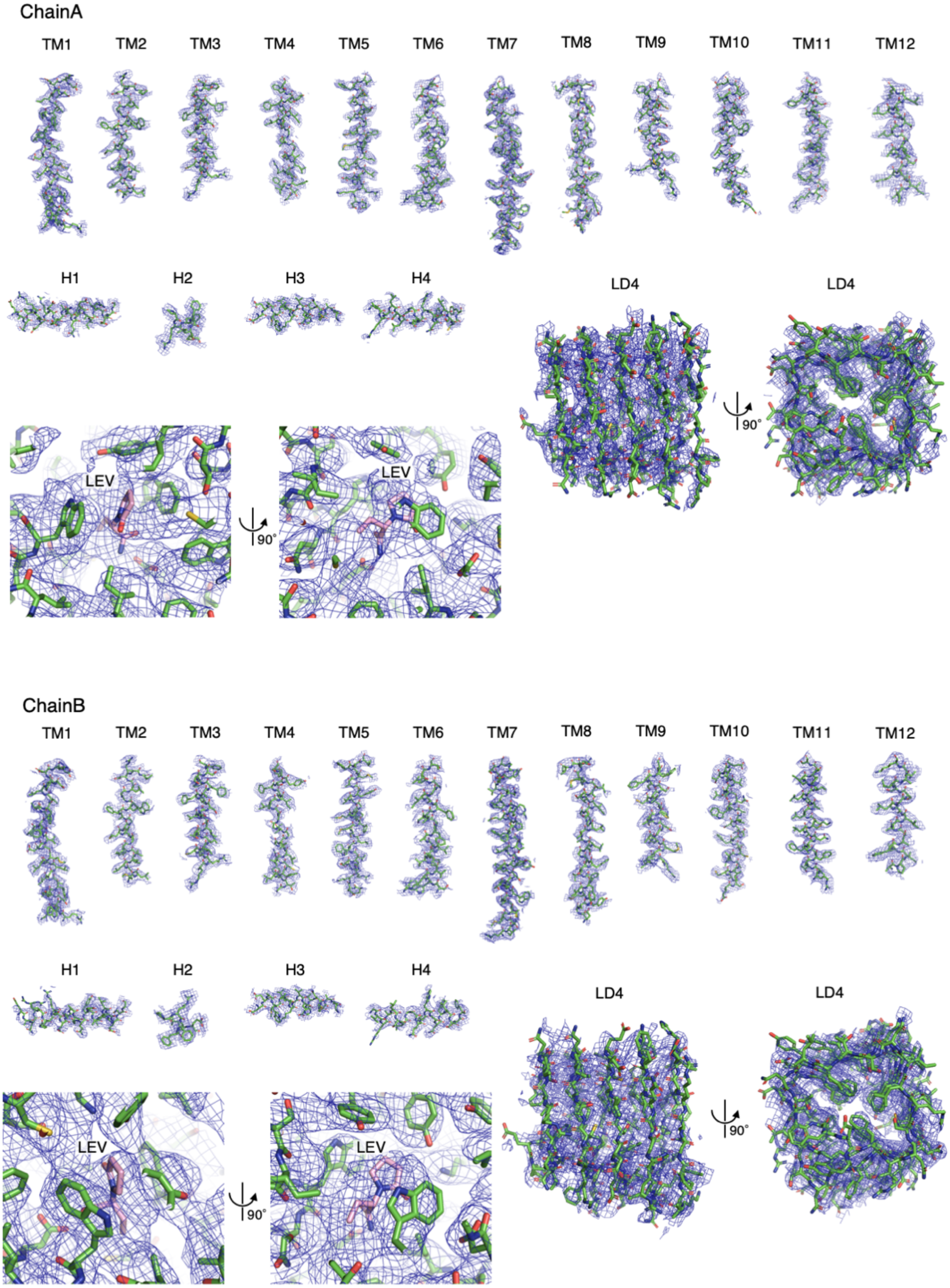

Supplementary Figure 9. Density maps for the SV2A–LEV dimer

Cryo-EM densities for each secondary structure segments of SV2A TMD and those for LD4A. Cryo-EM densities for LEV with the surrounding residues, viewed from two different angles, are also shown.

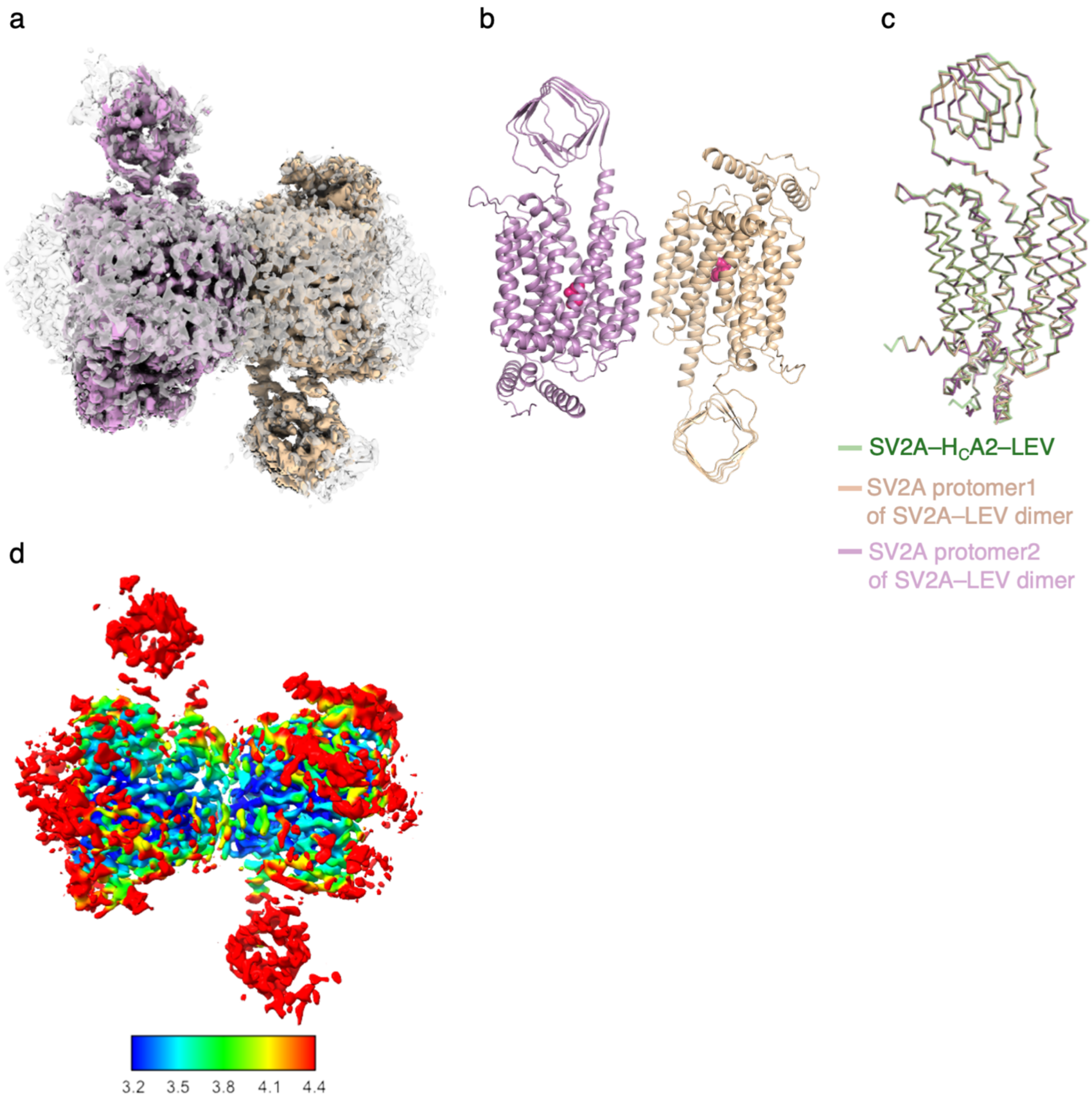

Supplementary Figure 10. Cryo-EM structure of the SV2A–LEV dimer

a. Cryo-EM map of the SV2A–LEV dimer. Each protomer is colored in magenta and beige, respectively. **b.** Structure of the SV2A–LEV dimer. Coloring scheme is the same as that in **(a)**. The bound LEVs are shown in spheres (hot pink). **c.** Superposition of each SV2A protomer from the SV2A–LEV dimer and the SV2A–H$_C$A2–LEV complex. **d.** Local resolution maps of the SV2A–LEV dimer.

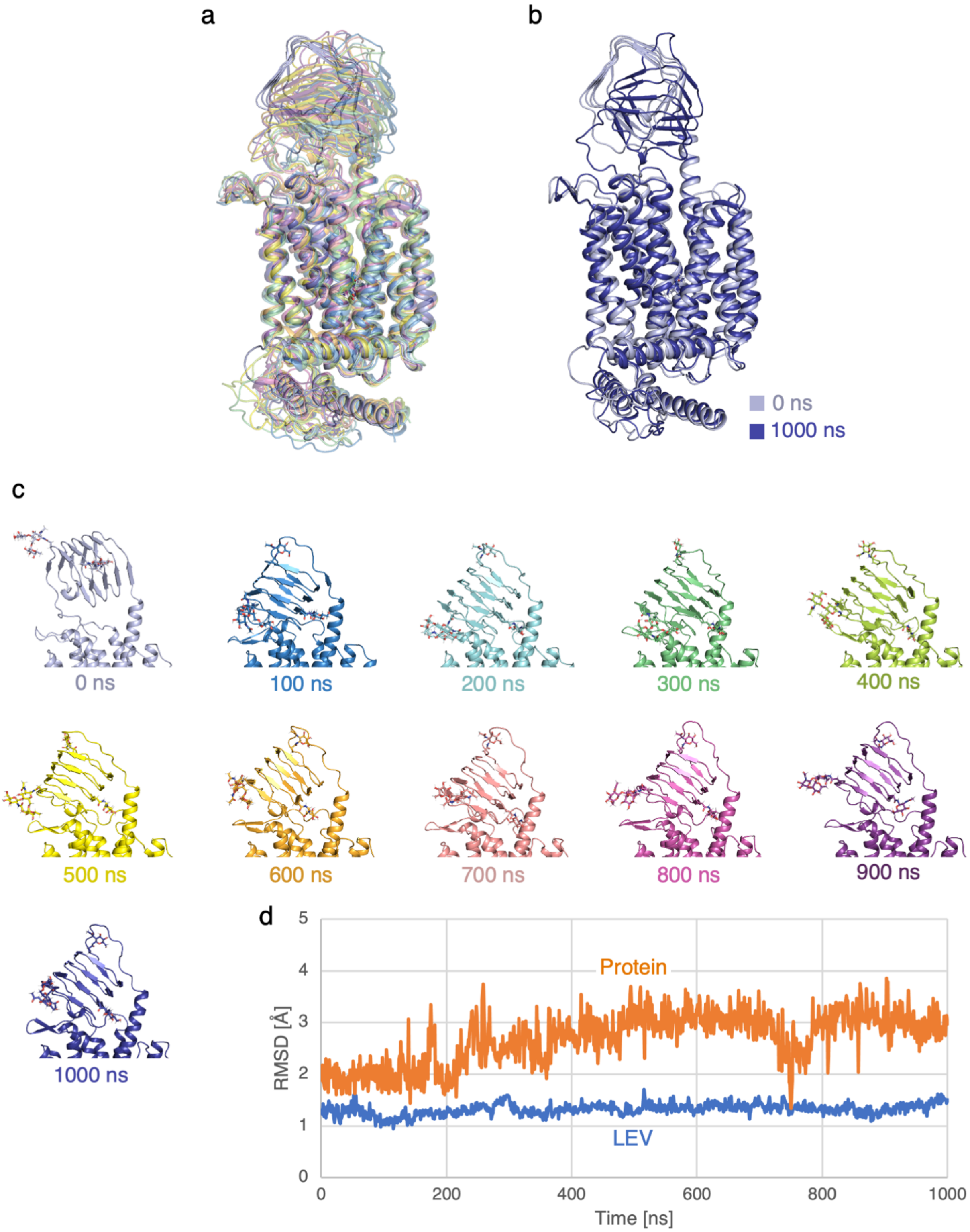

Supplementary Figure 11. MD simulations of the SV2A–LEV complex

a. Overlay of the individual MD simulation of the SV2A–LEV complex every 100 ns. **b.** MD simulations of the SV2A–LEV complex at 100 ns (light purple) and at 1000 ns (deep blue). **c.** MD simulations of the LD4 domain every 100 ns. **d.** The rmsd of the protein and the bound LEV are plotted.

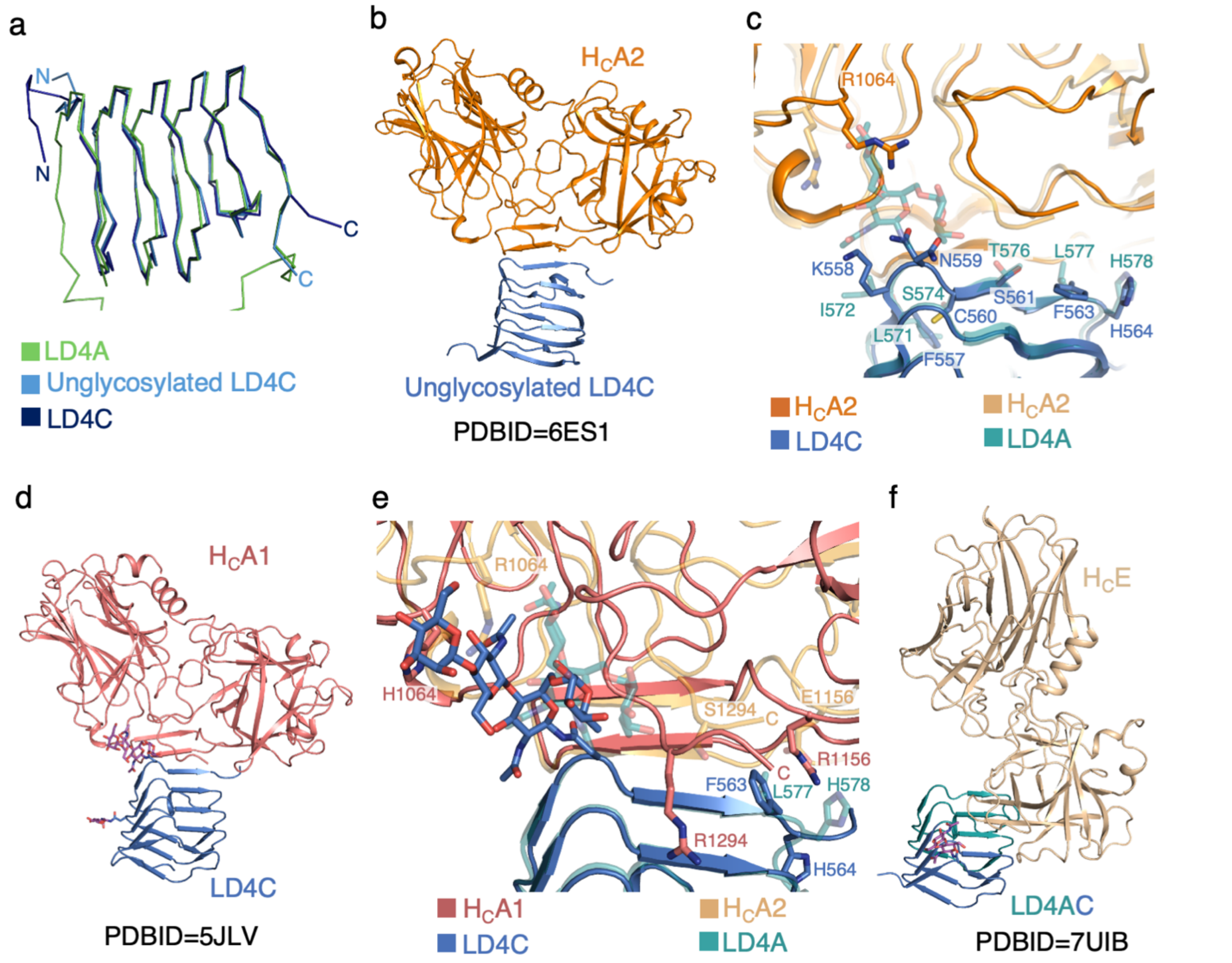

Supplementary Figure 12. Structural comparison with the other BoNT–SV2 LD4 complexes

a. Superposition of LD4Cs onto LD4A. **b.** Crystal structure of H_CA2–LD4C complex (PDBID=6ES1). LD4C is unglycosylated, as it was expressed in *E. coli*. **c.** Structural comparison of the binding interface between the H_CA2–LD4A and the H_CA2–LD4C complexes. **d.** Crystal structure of H_CA1–LD4C complex (PDBID=5JLV). SV2C LD4 is glycosylated, as it was expressed using HEK293 cells. **e.** Structural comparison of the binding interface between the H_CA2–SV2A and the H_CA1–LD4C complexes. **f.** Crystal structure of the H_CE–LD4AC (PDBID=7UIB).

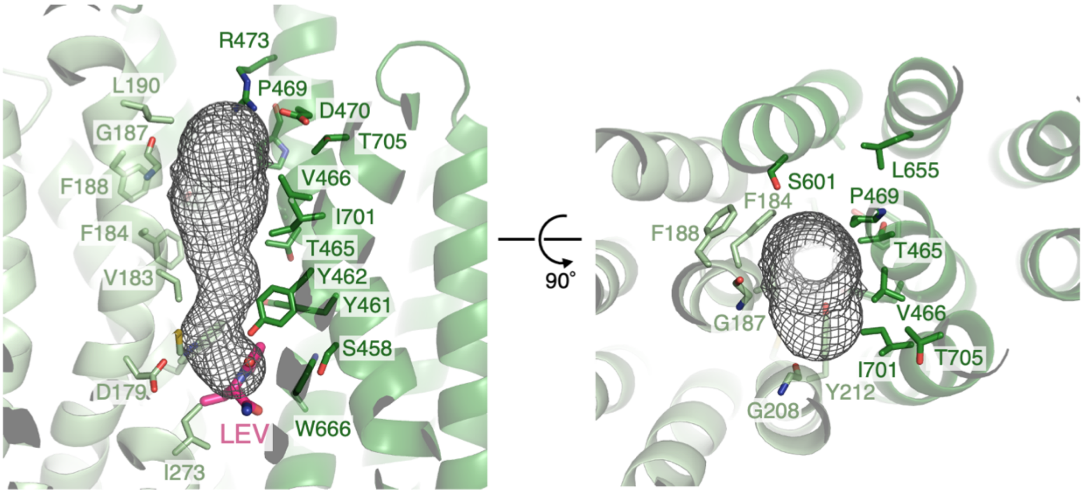

Supplementary Figure 13. The access tunnel to the LEV-binding pocket.

The access tunnel to the LEV-binding pocket is shown in gray mesh, viewed from the side (left) and top (right). The residues surrounding the tunnel are shown in sticks.

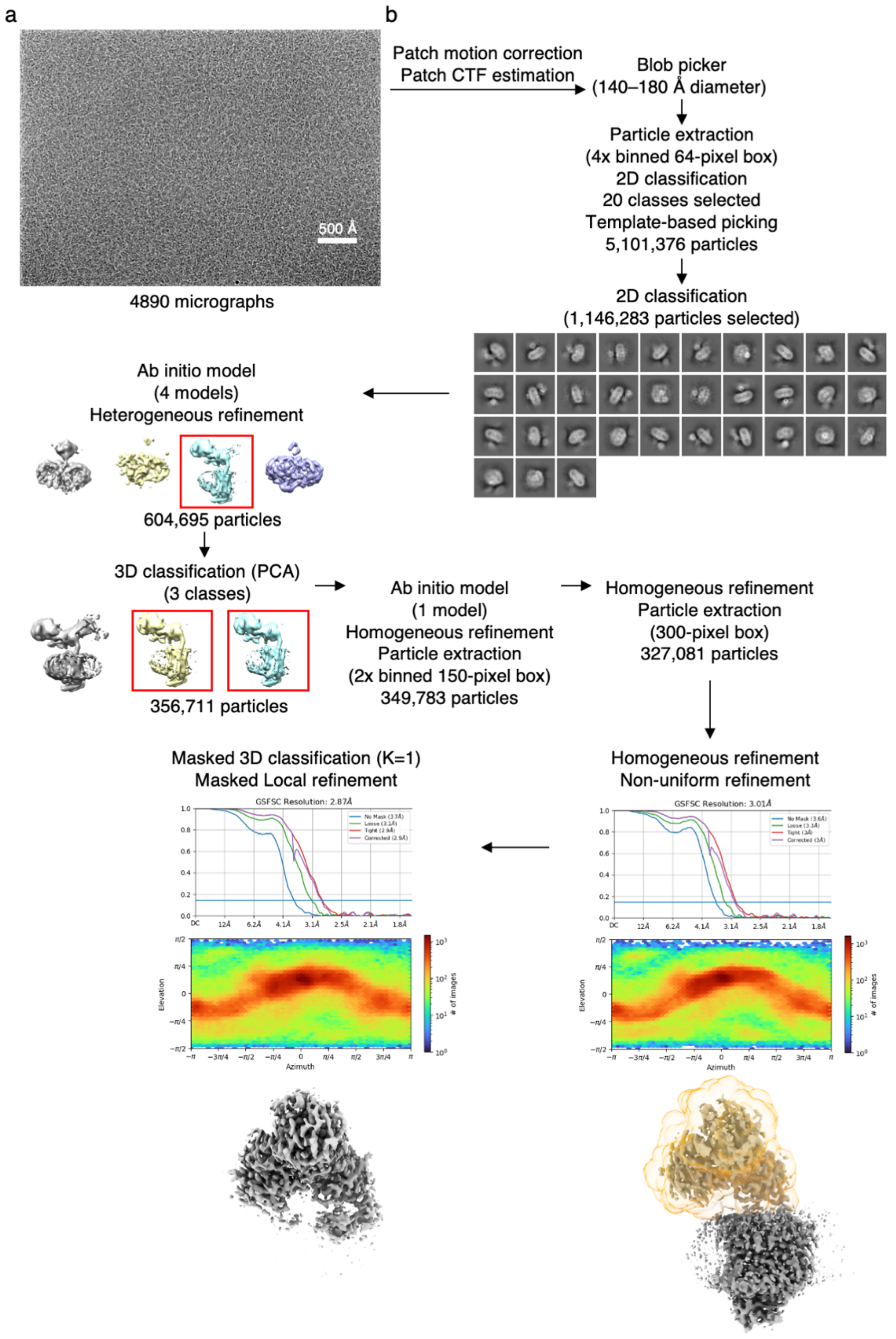

Supplementary Figure 14. Cryo-EM data and processing for the SV2A–H$_C$A2 complex

a. A representative micrograph of the SV2A–H$_C$A2 complex. We made at least two vitrified grids with similar particle images, and the micrographs were collected from a single grid. **b.** A flow chart for data processing and the final maps with Euler angle distribution plots.

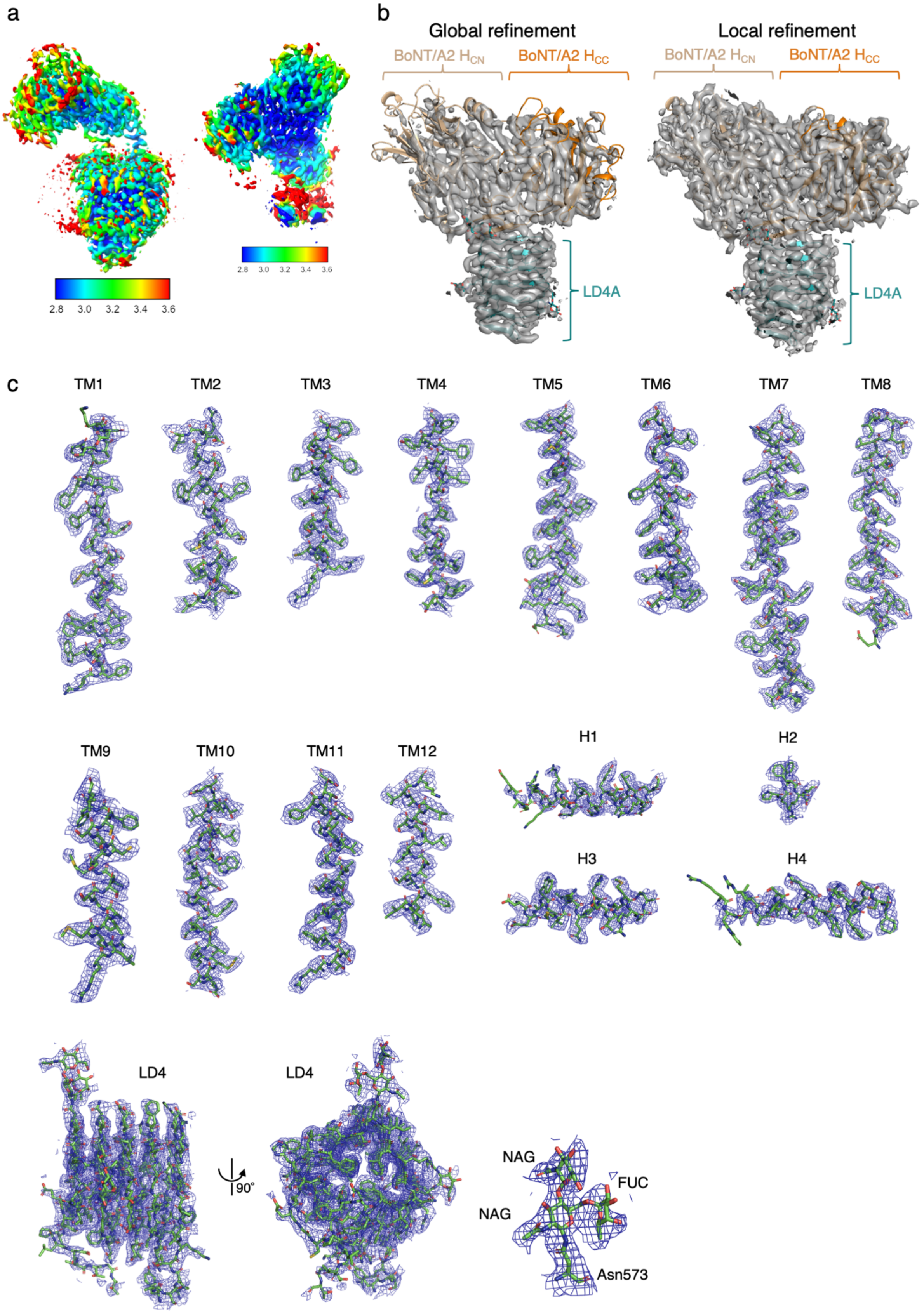

Supplementary Figure 15. Density maps for the SV2A–H$_C$A2 complex

a. Local resolution maps of the global refinement map (SV2A–H$_C$A2) and the local refinement map (LD4A–H$_C$A2). **b.** The cryo-EM density of the global refinement map covering the LD4A–H$_C$A2 region, and that of the local refinement map. **c.** Cryo-EM densities for each secondary structure segments of SV2A TMD and those for LD4A.

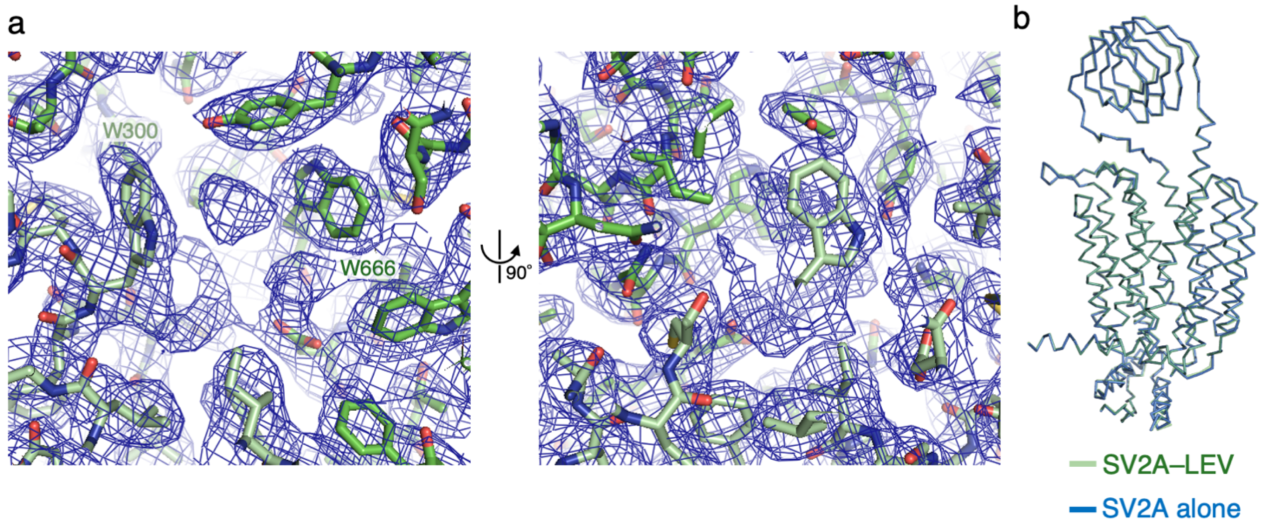

Supplementary Figure 16. Cryo-EM structure of the SV2A–H$_C$A2 complex

a. The cryo-EM density map of the LEV-binding site in the SV2A–H$_C$A2 complex. **b.** Superposition of SV2As from the SV2A–H$_C$A2 complexes with/without LEV.

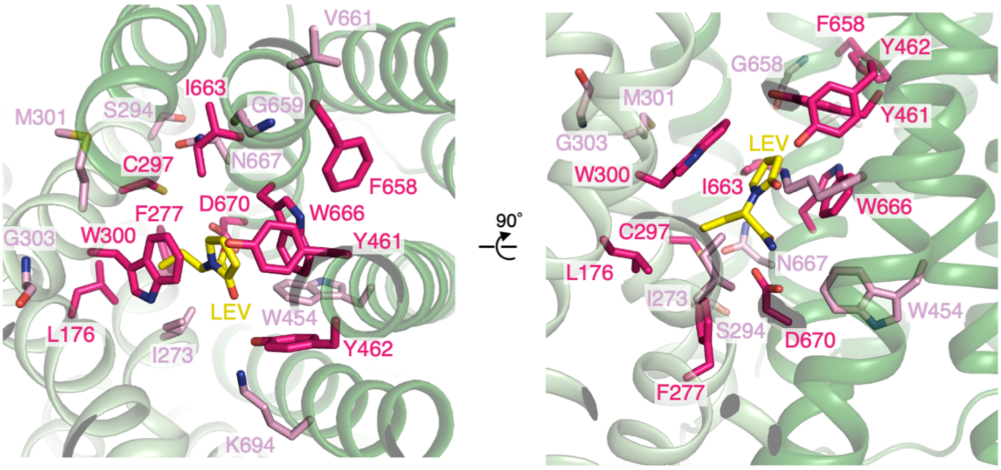

Mutation	Effect on Binding	Reference
I273A	reduced	Shi J., et al. 2011
F277A	reduced	Shi J., et al. 2011
F277A	severely reduced	Wood MD., et al 2018
S294A	reduced	Shi J., et al. 2011
C297 alkylated	abolished	Shi J., et al. 2011
W300A	abolished	Shi J., et al. 2011
W300F	reduced	Shi J., et al. 2011
W300F	severely reduced	Wood MD., et al 2018
M301A	reduced	Shi J., et al. 2011
G303A	reduced	Shi J., et al. 2011
W454A	reduced	Lee J., et al 2015
Y462A	reduced	Shi J., et al. 2011
Y462A	severely reduced	Wood MD., et al 2018
F658A	reduced	Shi J., et al. 2011
F658A	severely reduced	Wood MD., et al 2018
G659A	reduced	Shi J., et al. 2011
V661A	reduced	Shi J., et al. 2011
V661A	reduced	Wood MD., et al 2018
I663A	abolished	Shi J., et al. 2011
I663A	severely reduced	Wood MD., et al 2018
W666A	severely reduced	Shi J., et al. 2011
W666A	severely reduced	Wood MD., et al 2018
N667A	reduced	Shi J., et al. 2011
D670A	severely reduced	Wood MD., et al 2018
D670A	abolished	Lee J., et al 2015
K694A	reduced	Shi J., et al. 2011

Supplementary Figure 17. Mapping of the residues previously identified as key residues for binding to racetams.

The residues identified as key residues for binding to racetams by the previous mutational analyses are mapped on the cryo-EM structure. The residues, whose mutation abolished or severely reduced binding, are colored in hot pink. The residues, whose mutation reduced binding, are colored in pink. Each residue is listed below.

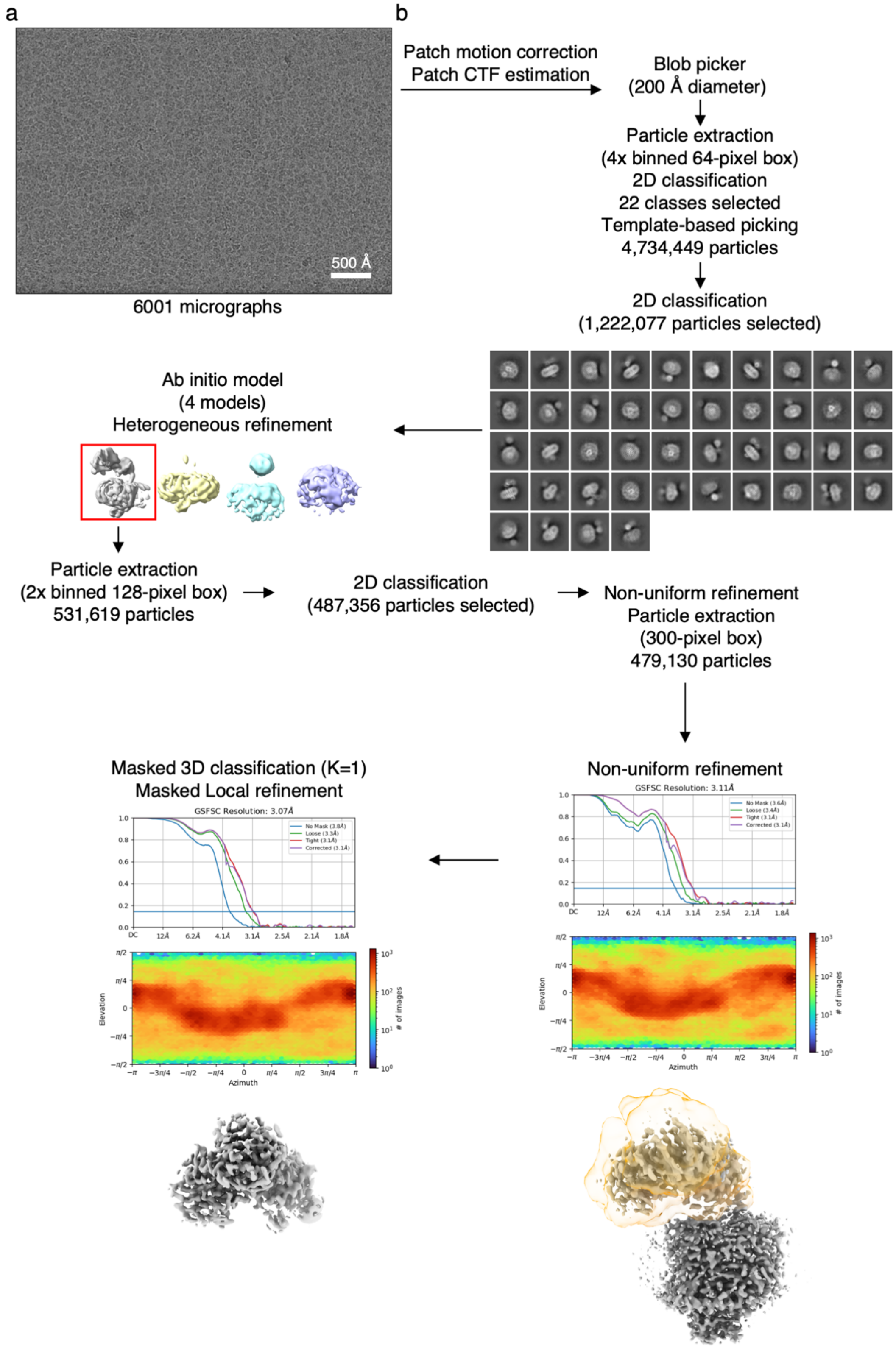

Supplementary Figure 18. Cryo-EM data and processing for the SV2A–H$_C$A2–BRV complex

a. A representative micrograph of the SV2A–H$_C$A2–BRV complex. We made at least two vitrified grids with similar particle images, and the micrographs were collected from a single grid. **b.** A flow chart for data processing and the final maps with Euler angle distribution plots.

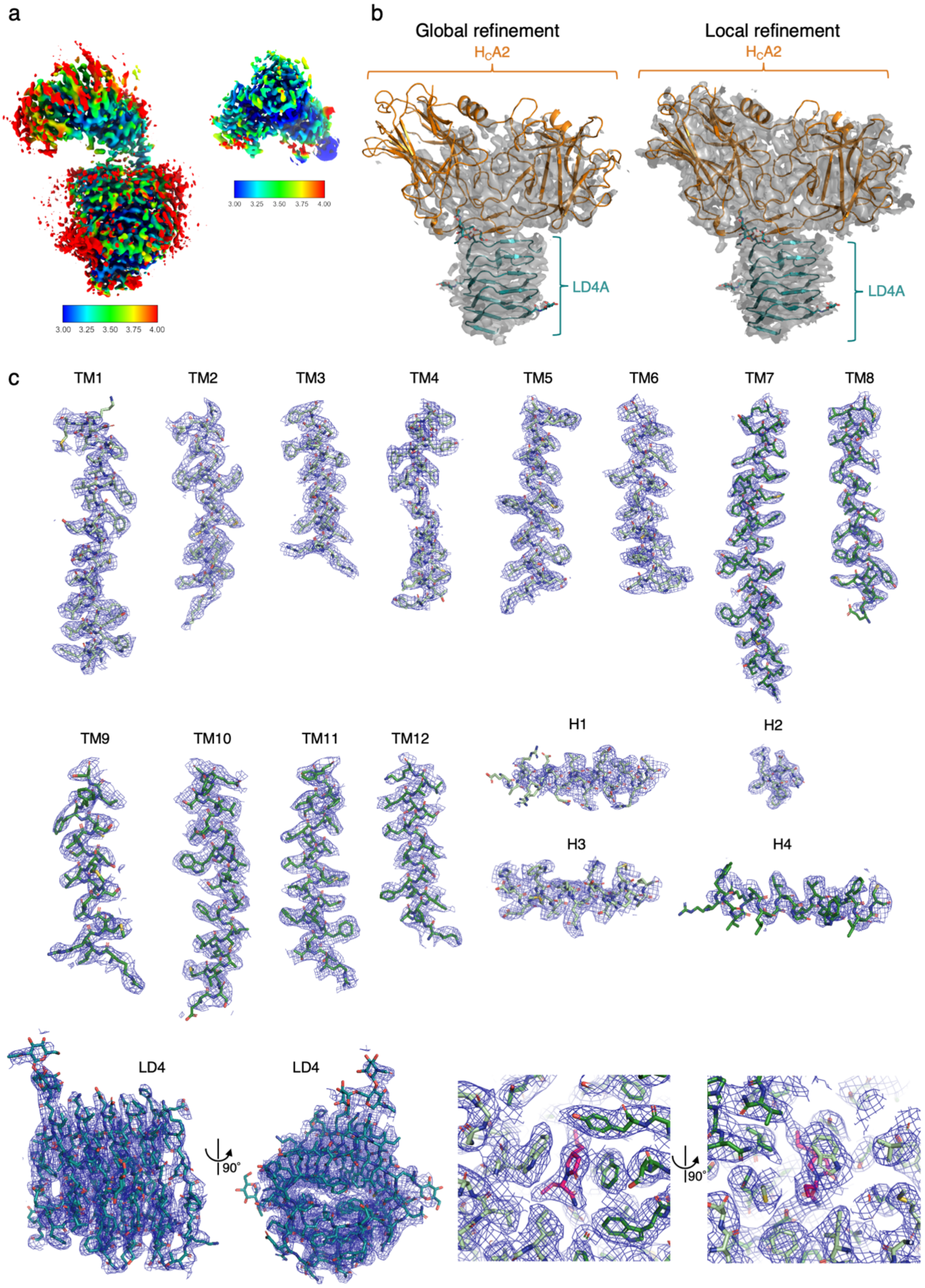

Supplementary Figure 19. Density maps for the SV2A–H$_{C}$A2–BRV complex

a. Local resolution maps of the global refinement map (SV2A–H$_{C}$A2–BRV) and the local refinement map (LD4A–H$_{C}$A2). **b.** The cryo-EM density of the global refinement map covering the LD4A–H$_{C}$A2 region, and that of the local refinement map. **c.** Cryo-EM densities for each secondary structure segments of SV2A TMD and those for LD4A. Cryo-EM densities for LEV with the surrounding residues, viewed from two different angles, are also shown. Cryo-EM densities for BRV with the surrounding residues, viewed from two different angles, are also shown.

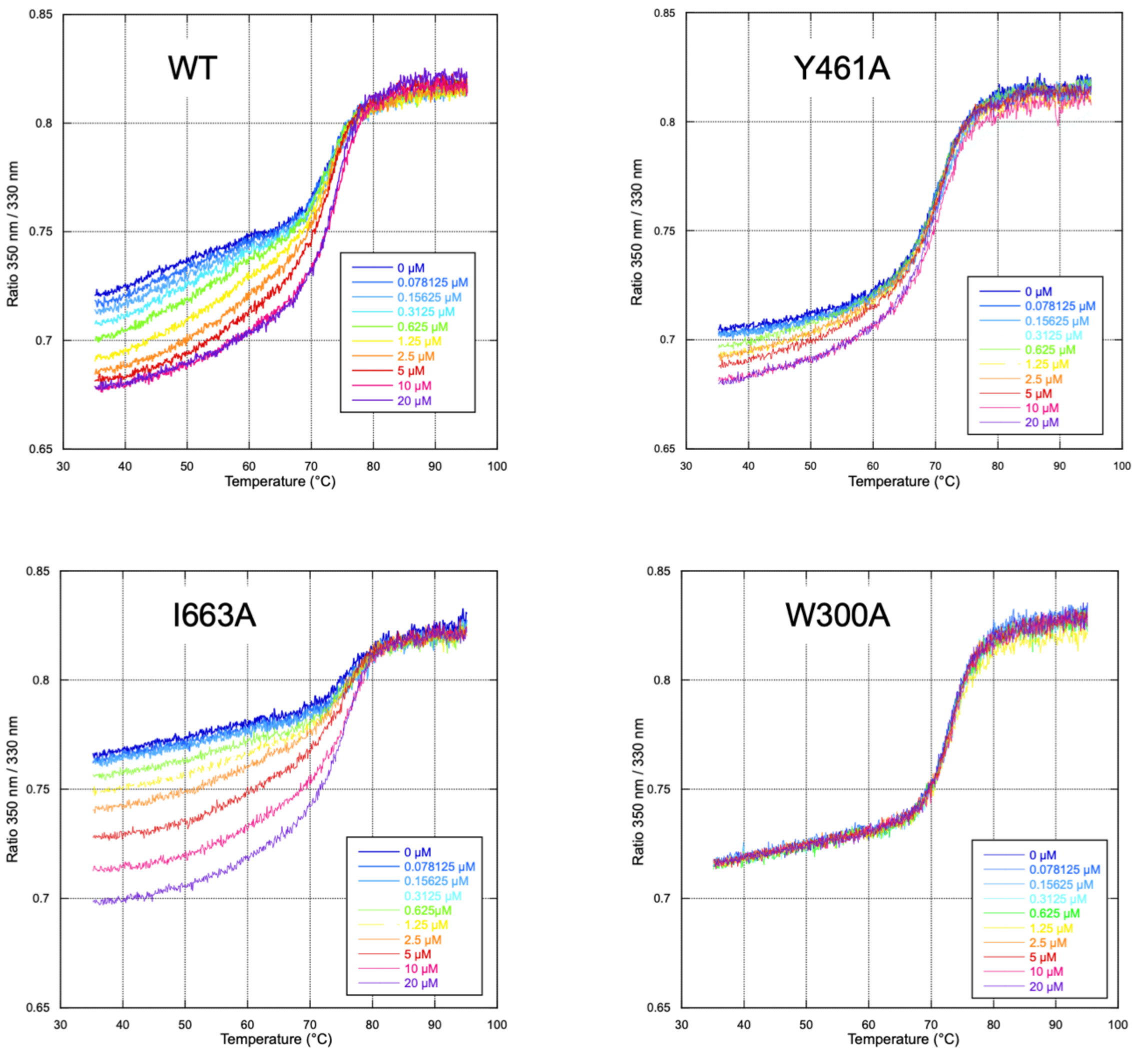

Supplementary Figure 20. The spectral shift of the intrinsic tryptophan fluorescence and thermal shift curve.

The ratio of the intrinsic tryptophan fluorescence at 350 nm/330 nm was measured using Tycho NT.6 (Nanotemper). The temperature was increased from 35 °C to 95 °C to obtain the thermal shift curve. The thermal shift curves of SV2A with a given concentration of BRV are shown in rainbow colors. The initial ratio (35.2°C) at a given concentration of BRV was used to obtain a dose-response curve (Fig. 4b). The experiments were repeated three times with similar results. Source data are provided as a Source Data file.

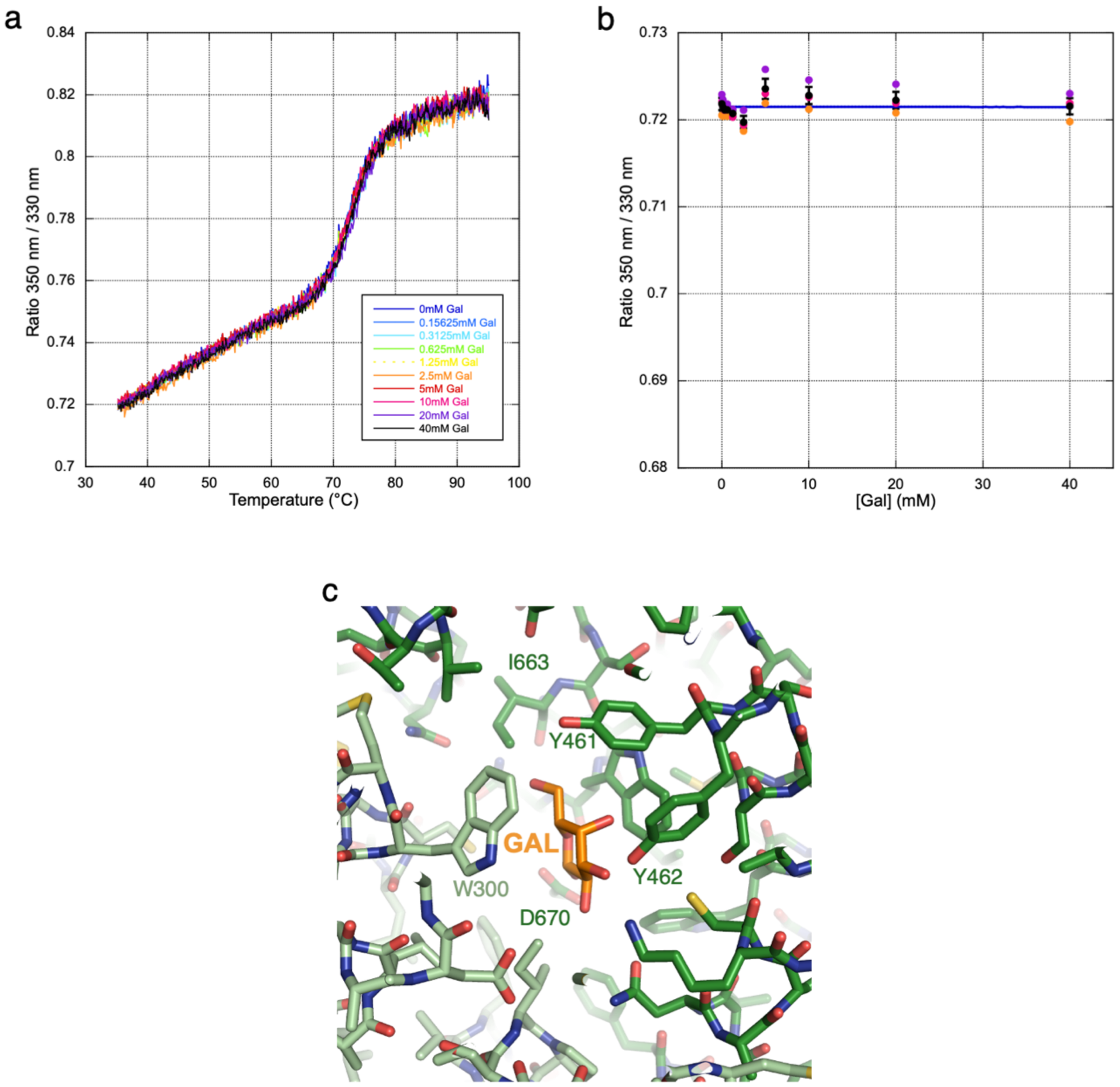

Supplementary Figure 21. Galactose-binding of SV2A

a. The label-free spectral shift assay of SV2A with galactose. The thermal shift curves of SV2A at a given concentration of galactose (Gal) are shown. Source data are provided as a Source Data file. **b.** Initial ratios (35.2 °C) from **(a)** at a given concentration of Gal are plotted. Prots (black) show the mean derived from n=3 technical replicates (red, orange, and purple circles) and the error bars show the standard deviations. Source data are provided as a Source Data file. **c.** Manual docking model of the galactose-bound SV2A. Galactose was manually docked in the LEV-binding pocket in the SV2A structure with no steric hindrance.

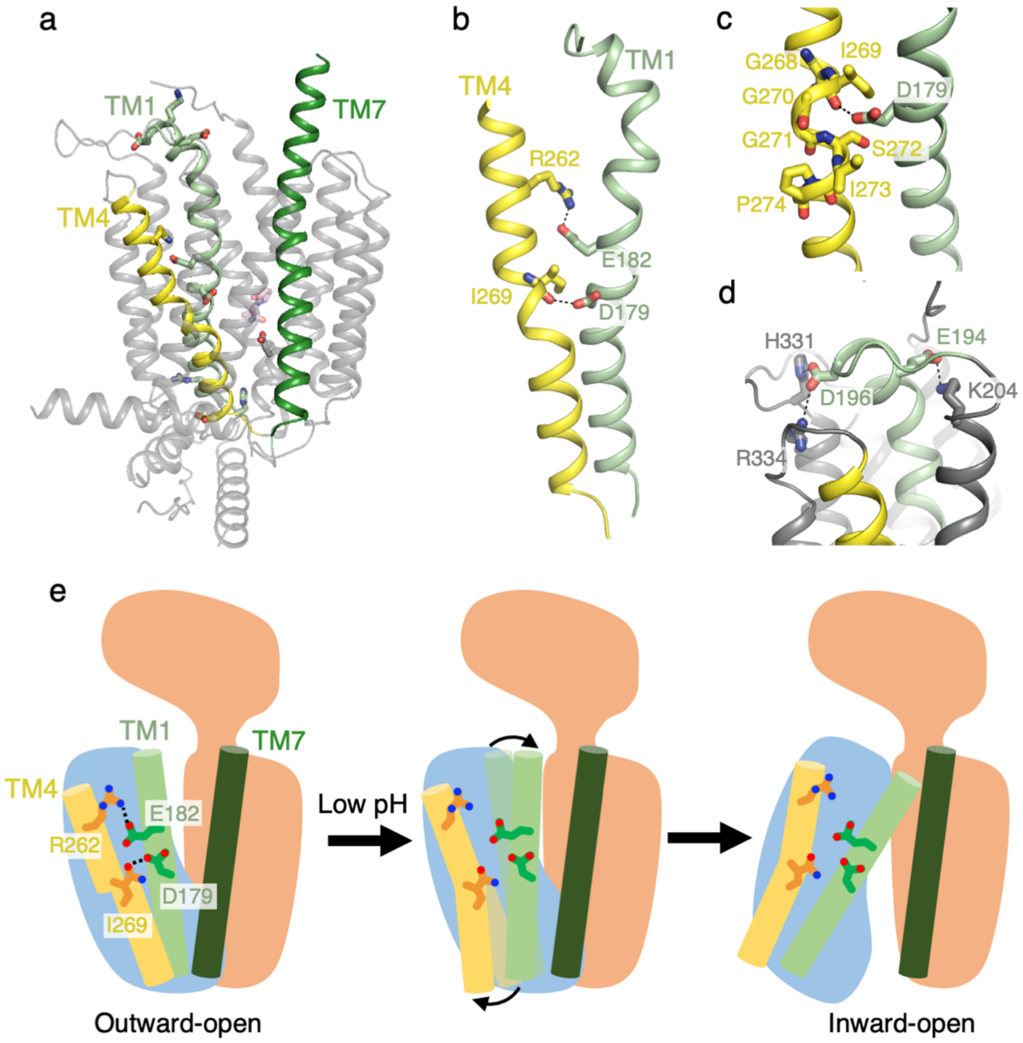

Supplementary Figure 22. The hypothesized proton-driven transport model

a. Asp, Glu, His, Arg, and Lys residues in the SV2A TMD. For clarity, those in the cytoplasmic domain and the whole region of LD4 are omitted. **b.** TM1–TM4 interaction through the hydrogen bonds. **c.** Asp179 appears to stabilize the intrinsic flexible region (Gly268–Pro274) on TM4. **d.** Hydrogen bond network of the vesicular lumen region of the TM1. **e.** The hypothesized model of the proton-driven conformational transition.

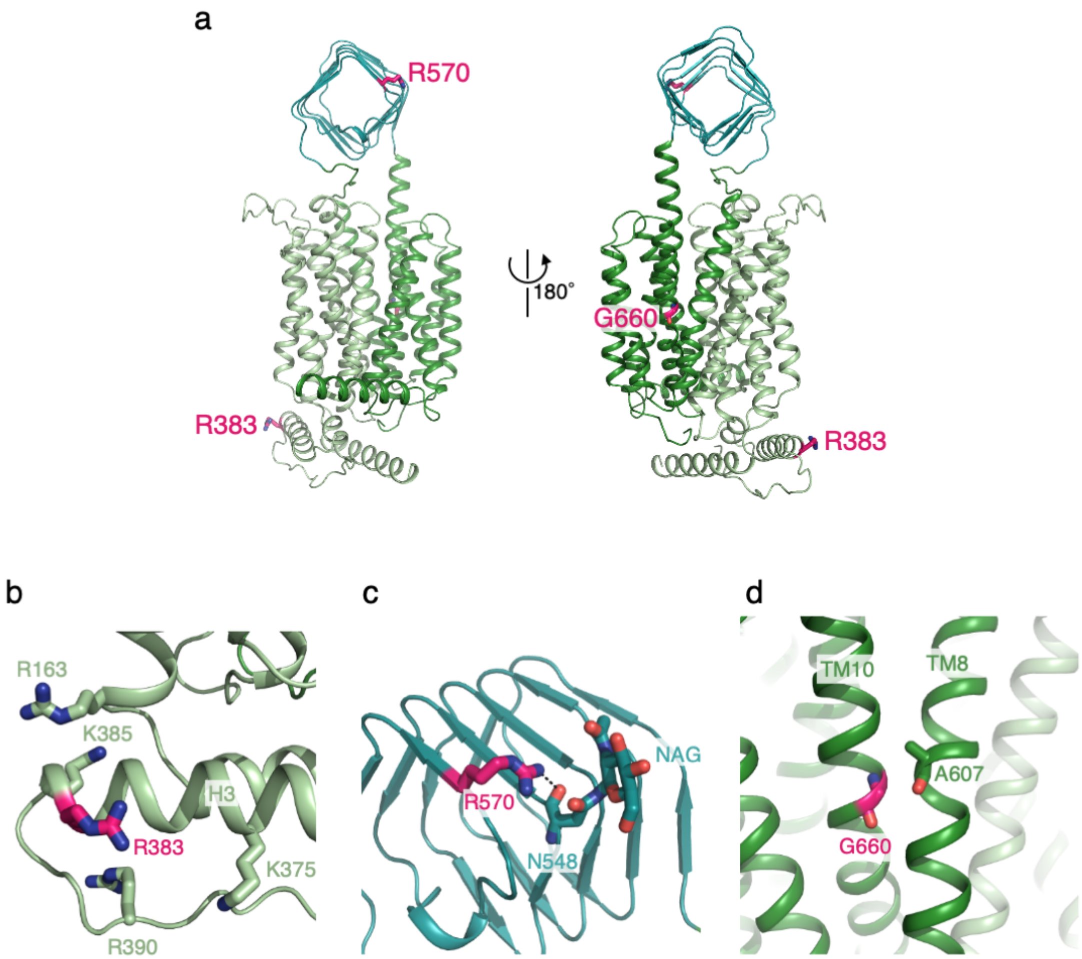

Supplementary Figure 23. Pathogenic mutations of SV2A

a. Three pathogenic mutation sites are mapped onto the SV2A structure. **b.** Closed-up view of Arg383 and neighboring positively charged residues. **c.** Closed-up view of Arg570 and Asn548 with the attached N-glycan. **d.** Closed-up view of Gly660 and the neighboring TM8.

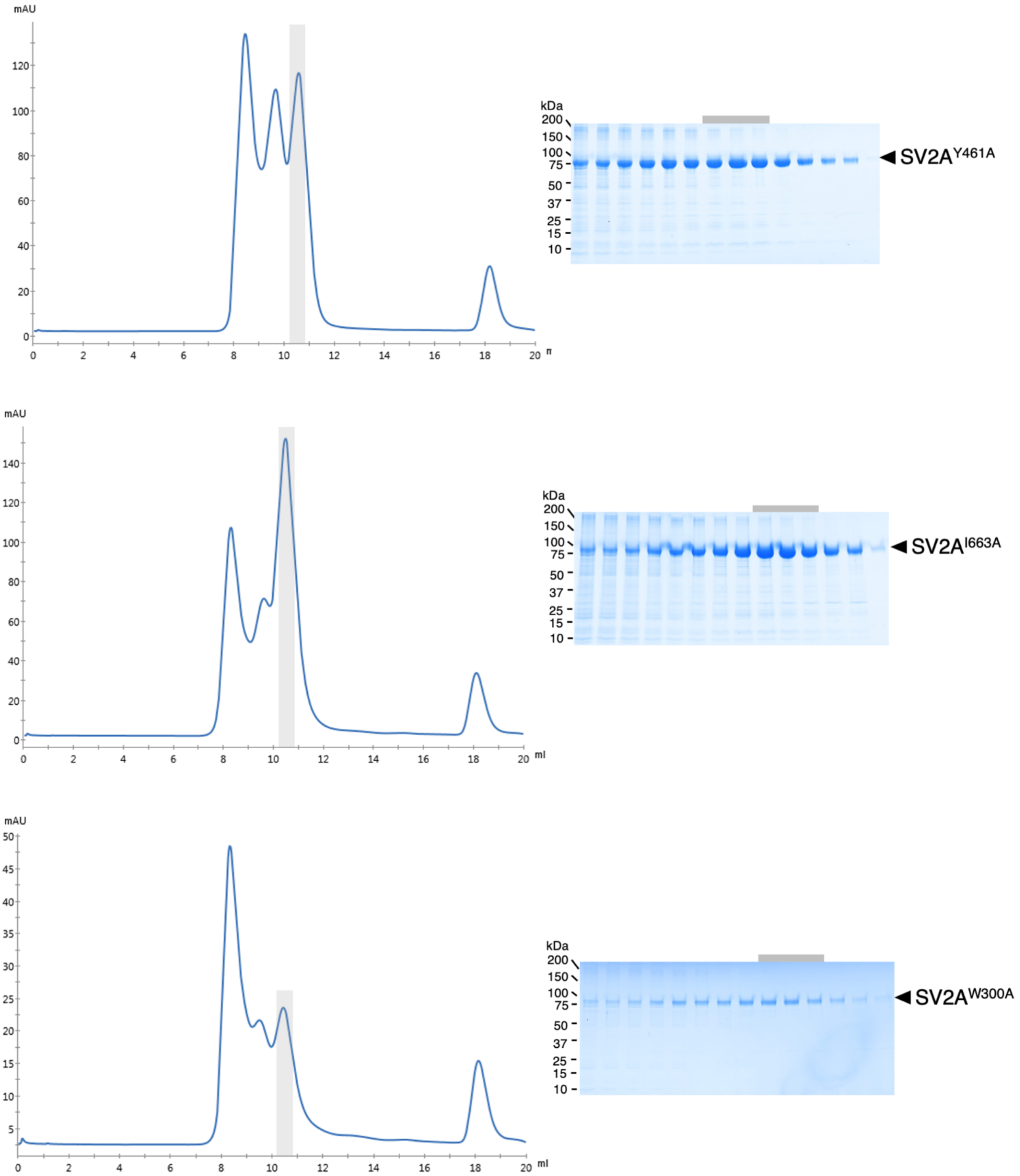

Supplementary Figure 24. Purification of SV2A^{Y461A}, SV2A^{I663A}, and SV2A^{W300A}

Size exclusion chromatography (SEC) profiles and the corresponding SDS-PAGE gels of SV2A^{Y461A}, SV2A^{I663A}, and SV2A^{W300A}. The shadowed areas were pooled and used for the spectral shift assay (Fig. 4b). Experiments were repeated twice independently with similar results.

Extended Data Figure 1

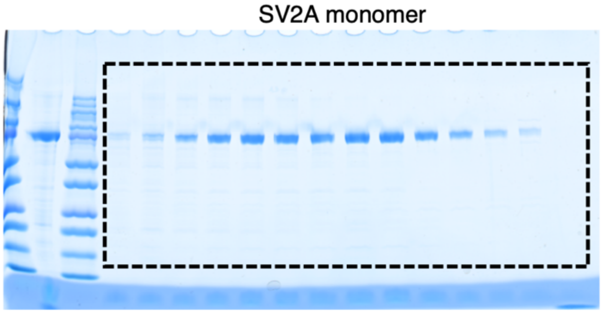

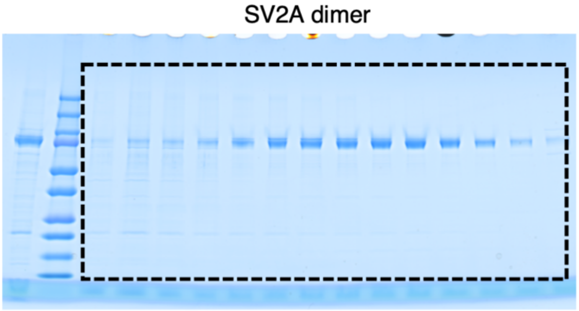

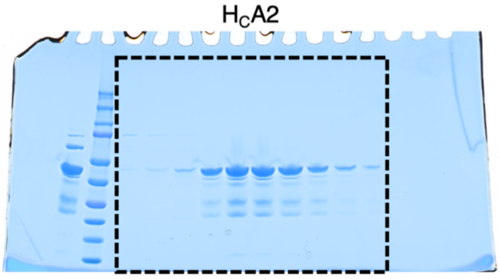

Extended Data Figure 23

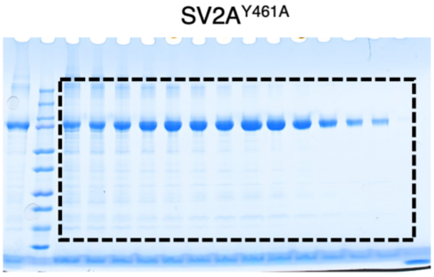

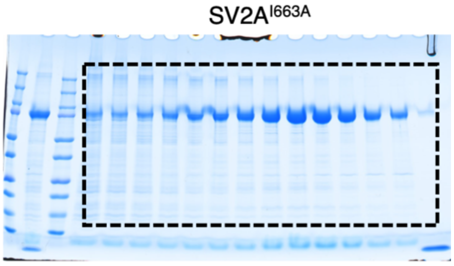

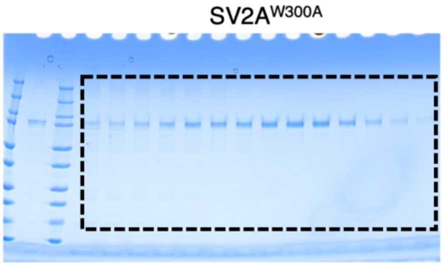

Extended Data Figure 7

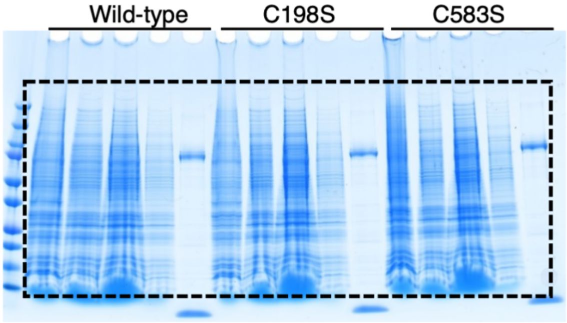

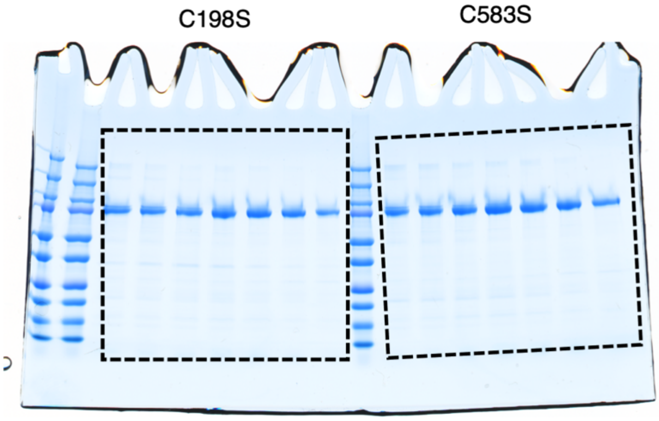

Supplementary Figure 25. Uncropped images of gels

Experiments were repeated twice independently with similar results.

Supplementary Table1. Cryo-EM data

	SV2A–HCA2–LEV				SV2–LEV	SV2A–HCA2			SV2A–HCA2–BRV		
	**Global**	**Local**	**Composite**	**dimeric complex**		**Global**	**Local**	**Composite**	**Global**	**Local**	**Composite**
EMDB	36392	36394	36616	36397	36398	36395	36396	36617	36933	36934	36935
PDB	8JLC	8JLE	8JS8	8JLH	8JLI	8JLF	8JLG	8JS9			8K77
Data collection and processing											
Magnification	105,000				105,000	105,000			105,000		
Voltage (kV)	300				300	300			300		
Total dose (e^-/Å ^{2})	50.8				50.2	50.0			50.0		
Defocus range (μm)	0.8 – 2.0				0.8 - 2.0	0.8 - 2.0			0.8 - 2.0		
Pixel size (Å)	0.83				0.83	0.83			0.83		
Initial particle images (no.)	6,409,122			6,409,122	6,435,573	5,101,376			4,734,449		
Final particle images (no.)	646,723	646,723		425,512	424,198	327,081	327,081		479,130	479,130	
Map resolution (Å)	2.88	2.82		2.90	3.38	3.01	2.87		3.11	3.07	
FSC threshold	0.143	0.143		0.143	0.143	0.143	0.143		0.143	0.143	
Map resolution range	2.8–3.6	2.8–3.6		2.8–3.6	3.2–4.4	2.8–3.6	2.8–3.6		3.0–4.0	3.0–4.0	
Refinement											
Initial model used	8JLE	6ES1	8LLC	8JLC	8JLC	8JLC	8JLE	8JS8			8JS8
Model resolution (Å)											
FSC0.143, unmasked/masked	2.88/2.84	2.83/2.79	2.86/2.81	2.90/2.86	3.38/3.34	3.00/2.95	2.90/2.84	2.95/2.89			3.03/3.07
Map sharpening B factors (Å^2)	-131.0	-131.0		-105.6	-104.5	-128.6	-122.9		-143.5	-140.8	
Model composition											
Non-hydrogen atoms	8189	4408	8189	12624	9248	8177	4426	8177			8192
Protein residues	1012	528	1012	1554	1172	1012	531	1012			1012
Ligands	6	5	6	11	2	5	5	5			6
B factors (Å^2)											
Protein (Å^2)	196.82	129.93	63.47	164.42	186.78	179.61	115.55	65.52			63.47
Ligands (Å^2)	234.39	174.32	86.00	158.59	262.44	259.58	187.16	95.00			92.33
RMS deviation											
Bond length (Å)	0.010	0.010	0.003	0.010	0.008	0.011	0.011	0.004			0.007
Bond angle (°)	1.268	1.268	0.589	1.334	1.140	1.235	1.247	0.533			0.967
Molprobity score	1.78	2.01	1.87	1.86	1.70	1.65	1.78	1.59			1.70
Clash score	10.38	13.39	7.49	10.74	10.40	8.11	8.21	7.13			9.33
Rotamer outliers (%)	0	0	0	0.15	0	0	0	0			0
Ramachandran plot											
Favored (%)	96.32	94.47	96.92	95.50	97.08	96.72	95.26	96.82			96.72
Allowed (%)	3.68	5.34	3.08	4.37	2.92	3.28	4.74	3.18			3.28
Outlier (%)	0	0.19	0	0.13	0	0	0	0			0
