# Supplementary material for: Structural basis for antiepileptic drugs and botulinum neurotoxin recognition of SV2A
Source: Nat Commun. 2024 Apr 18;15:3027. doi: 10.1038/s41467-024-47322-4 (PMC11026379; doi:10.1038/s41467-024-47322-4)
Supplement: Supplementary file 2 — Reporting Summary [file 41467_2024_47322_MOESM2_ESM.pdf]

nature portfolio

Corresponding author(s): Atsushi Yamagata
Last updated by author(s): Mar 12, 2024

Reporting Summary

Nature Portfolio wishes to improve the reproducibility of the work that we publish. This form provides structure for consistency and transparency in reporting. For further information on Nature Portfolio policies, see our Editorial Policies and the Editorial Policy Checklist.

Statistics

For all statistical analyses, confirm that the following items are present in the figure legend, table legend, main text, or Methods section.

n/a	Confirmed	
☐	☒	The exact sample size (*n*) for each experimental group/condition, given as a discrete number and unit of measurement
☐	☒	A statement on whether measurements were taken from distinct samples or whether the same sample was measured repeatedly
☐	☒	The statistical test(s) used AND whether they are one- or two-sided<br>*Only common tests should be described solely by name; describe more complex techniques in the Methods section.*
☒	☐	A description of all covariates tested
☒	☐	A description of any assumptions or corrections, such as tests of normality and adjustment for multiple comparisons
☐	☒	A full description of the statistical parameters including central tendency (e.g. means) or other basic estimates (e.g. regression coefficient) AND variation (e.g. standard deviation) or associated estimates of uncertainty (e.g. confidence intervals)
☐	☒	For null hypothesis testing, the test statistic (e.g. *F*, *t*, *r*) with confidence intervals, effect sizes, degrees of freedom and *P* value noted<br>*Give P values as exact values whenever suitable.*
☒	☐	For Bayesian analysis, information on the choice of priors and Markov chain Monte Carlo settings
☒	☐	For hierarchical and complex designs, identification of the appropriate level for tests and full reporting of outcomes
☒	☐	Estimates of effect sizes (e.g. Cohen's *d*, Pearson's *r*), indicating how they were calculated

Our web collection on statistics for biologists contains articles on many of the points above.

Software and code

Policy information about availability of computer code

Data collection	Thermofisher EPU (ver 2.9)
Data analysis	cryoSPARC 4.2.1, Coot 0.9.8.1, Phenix 1.20.1, CCPEM-1.6.0, Refmac-5.8.0352, Servalcat-0.2.85, eLBOW, MolProbity, PISA-2.1.0, Caver-3.0.3 ChimeraX-1.2.5, PyMol 2.5.2, OPM database and PPM server, CHARMM and CHARMM-GUI server, GROMACS 2022, LINCS algorithm, MASCOT-2.8, Proteome Discoverer-3.0, Kaleidagraph 5 Mac

For manuscripts utilizing custom algorithms or software that are central to the research but not yet described in published literature, software must be made available to editors and reviewers. We strongly encourage code deposition in a community repository (e.g. GitHub). See the Nature Portfolio guidelines for submitting code & software for further information.

Data

Policy information about availability of data

All manuscripts must include a data availability statement. This statement should provide the following information, where applicable:

- Accession codes, unique identifiers, or web links for publicly available datasets
- A description of any restrictions on data availability
- For clinical datasets or third party data, please ensure that the statement adheres to our policy

The cryo-EM maps have been deposited in the Electron Microscopy Data Bank under accession numbers EMD-36392 [https://www.ebi.ac.uk/pdbe/entry/emdb/EMD-36392] (SV2A–HCA2–LEV), EMD-36394 [https://www.ebi.ac.uk/pdbe/entry/emdb/EMD-36394] (local refinement map of EMD-36392), EMD-36616 [https://www.ebi.ac.uk/pdbe/entry/emdb/EMD-36616] (composite map of EMD-36392 and EMD-36394), EMD-36397 [https://www.ebi.ac.uk/pdbe/entry/emdb/

EMD-36397] (SV2A–HCA2–LEV (dimeric complex)), EMD-36398 [https://www.ebi.ac.uk/pdbe/entry/emdb/ EMD-36398] (SV2A¬–LEV), EMD-36395 [https://www.ebi.ac.uk/pdbe/entry/emdb/EMD-36395] (SV2A–HCA2), EMD-36396 [https://www.ebi.ac.uk/pdbe/entry/emdb/ EMD-36396] (local refinement map of EMD-36395), EMD-36617 [https://www.ebi.ac.uk/pdbe/entry/emdb/EMD-36617] (composite map of EMD-36395 and EMD-36396), EMD-36933 [https://www.ebi.ac.uk/pdbe/entry/emdb/EMD-36933] (SV2A–HCA2–BRV), EMD-36934 [https://www.ebi.ac.uk/pdbe/entry/emdb/EMD-36934] (local refinement map of EMD-36933), EMD-36935 [https://www.ebi.ac.uk/pdbe/entry/emdb/EMD-36935] (composite map of EMD-36933 and EMD-36934). The coordinates have been deposited in the RCSB Protein Data Bank (PDB) accession codes 8JLC [http://doi.org/10.2210/pdb8JLC/pdb] (SV2A–HCA2–LEV for EMD-36392), 8JLE [http://doi.org/10.2210/pdb8JLE/pdb] (LD4A–HCA2 for EMD-36394), 8JS8 [http://doi.org/10.2210/pdb8JS8/pdb] (SV2A–HCA2–LEV for EMD-36616), 8JLH [http://doi.org/10.2210/pdb8JLH/pdb] (SV2A–HCA2–LEV (dimeric complex) for EMD-36397), 8JLI [http://doi.org/10.2210/pdb8JLI/pdb] (SV2A¬–LEV for EMD-36398), 8JLF [http://doi.org/10.2210/pdb8JLF/pdb] (SV2A–HCA2 for EMD-36395), 8JLG [http://doi.org/10.2210/pdb8JLG/pdb] (LD4A–HCA2 for EMD-36396), 8JS9 [http://doi.org/10.2210/pdb8JS9/pdb] (SV2A–HCA2 for EMD-36617), 8K77 [http://doi.org/10.2210/pdb8K77/pdb] (SV2A–HCA2–BRV for EMD-36935), respectively. The mass spectrometry proteomics data have been deposited to the ProteomeXchange Consortium via the PRIDE [1] partner repository with the dataset identifier PXD050355 and 10.6019/PXD050355. Source data are provided with this paper.

Research involving human participants, their data, or biological material

Policy information about studies with human participants or human data. See also policy information about sex, gender (identity/presentation), and sexual orientation and race, ethnicity and racism.

Reporting on sex and gender	n/a
Reporting on race, ethnicity, or other socially relevant groupings	n/a
Population characteristics	n/a
Recruitment	n/a
Ethics oversight	n/a

Note that full information on the approval of the study protocol must also be provided in the manuscript.

Field-specific reporting

Please select the one below that is the best fit for your research. If you are not sure, read the appropriate sections before making your selection.

☒ Life sciences ☐ Behavioural & social sciences ☐ Ecological, evolutionary & environmental sciences

For a reference copy of the document with all sections, see nature.com/documents/nr-reporting-summary-flat.pdf

Life sciences study design

All studies must disclose on these points even when the disclosure is negative.

Sample size	No statistical method was performed to predetermine the sample size. The size of the cryo-EM data was based on the cryo-EM time allocation and previous knowledge estimating the size sufficient to achieve a high-resolution cryo-EM density map. For insect cell-based transport assay, four independent experiments were performed. A minimum number of the experiments was determined to obtain a reliable statistical results in consideration of time and labor power.
Data exclusions	No biochemical data have been excluded.
Replication	We purified the wild-type protein times and purified the mutant proteins at least twice. Label-free spectral shift assays were repeated three times with similar results. The other experiments were performed at least twice. All the experimental findings were reproduced.
Randomization	For cryo-EM structure determination, the data were randomly split into two groups to calculate the gold standard FSC correlation. For the other functional experiments, samples were not allocated to groups and thus randomization is not relevant for this study.
Blinding	Investigators were not blinded. An individual or group of people conducted each experiment according to their expertise. Blinding is not necessary for both cryo-EM structure determination and functional studies, because both experiments do not require subject assessment of data.

Reporting for specific materials, systems and methods

We require information from authors about some types of materials, experimental systems and methods used in many studies. Here, indicate whether each material, system or method listed is relevant to your study. If you are not sure if a list item applies to your research, read the appropriate section before selecting a response.

Materials & experimental systems

n/a	Involved in the study	
☒	☐	Antibodies
☐	☒	Eukaryotic cell lines
☒	☐	Palaeontology and archaeology
☒	☐	Animals and other organisms
☒	☐	Clinical data
☒	☐	Dual use research of concern
☒	☐	Plants

Methods

n/a	Involved in the study	
☒	☐	ChIP-seq
☒	☐	Flow cytometry
☒	☐	MRI-based neuroimaging

Eukaryotic cell lines

Policy information about cell lines and Sex and Gender in Research

Cell line source(s)	Sf9 cells were purchased from Thermofisher.
Authentication	No further authentication was performed to the cell lilne purchased from Thermo Fisher Scientific
Mycoplasma contamination	Mycoplasma contamination was not tested.
Commonly misidentified lines (See ICLAC register)	Commonly misidentified lines were not used in this study.
